# Impact of therapeutic exercises on pain-related outcomes in patients with knee osteoarthritis: an umbrella review of 116 systematic reviews

**DOI:** 10.3389/fpain.2026.1717540

**Published:** 2026-03-10

**Authors:** Dmitriy Viderman, Sultan Kalikanov, Akerke Mazhibiyeva, Alua Shagirova, Malika Toleubekova, Mina Aubakirova, Yerkin G. Abdildin

**Affiliations:** 1 Department of Surgery, School of Medicine, Nazarbayev University, Astana, Kazakhstan; 2 Department of Anesthesiology, Intensive Care, and Pain Medicine, National Research Oncology Center, Astana, Kazakhstan; 3 Department of Life Sciences, School of Sciences and Humanities, Nazarbayev University, Astana, Kazakhstan; 4 Department of Mechanical and Aerospace Engineering, School of Engineering and Digital Sciences, Nazarbayev University, Astana, Kazakhstan

**Keywords:** AMSTAR-2, exercise therapy, joint function, knee biomechanics, qigong, tai chi

## Abstract

**Background and objectives:**

Chronic knee pain is highly prevalent in various knee diseases. It significantly decreases patients’ quality of life. Among non-surgical interventions, exercise is considered a promising approach for alleviating chronic knee pain. This umbrella review aimed to systematically synthesize and critically appraise evidence from recent systematic reviews on the effectiveness of therapeutic exercises for knee osteoarthritis across pain, physical function, quality of life, and biomechanical outcomes.

**Materials and methods:**

We searched for systematic reviews with/without meta-analyses in PubMed, Scopus, and Cochrane Library databases.

**Results:**

A total of 116 systematic reviews and meta-analyses were included, published predominantly after 2014. Interrater agreement was substantial (*κ* = 0.76). Methodological quality was generally low, with 81% of reviews rated low or critically low by AMSTAR-2. Most reviews focused on knee osteoarthritis (72%) and evaluated strength/resistance, aerobic, or mind–body exercises. Pain was the most frequently reported outcome, with improvement noted in 69 reviews, followed by physical function (44 reviews) and quality of life (22 reviews). However, biomechanical outcomes were infrequently assessed and often inconclusive. Across outcomes, positive findings were largely derived from reviews with methodological limitations, warranting cautious interpretation. Thus, the percentages reflect the distribution of reported effects, and the magnitude and reliability of these effects remain limited.

**Conclusions:**

Therapeutic exercises may help reduce pain, improve physical function and quality of life in patients with knee osteoarthritis. However, the overall certainty of evidence is limited by the variable methodological quality of the included systematic reviews. At present, no single type of therapeutic exercise can be considered the most effective for all individuals with knee osteoarthritis.

**Systematic Review Registration:**

https://osf.io/pqgrv/overview, doi: 10.17605/OSF.IO/PQGRV.

## 1 Introduction

Patients with persistent knee pain present a substantial burden in the global health field, with increased expenditures and workload for medical facilities all around the world. It brings discomfort and can severely worsen a patient's quality of life. Knee pain may affect different groups, starting from older adults and ending with proficient athletes (1–3). Disorders such as knee osteoarthritis (KOA), chondromalacia patellae (CMP), patellar tendinopathy (PT), and patellofemoral pain (PFP) can contribute to the development of knee pain. Among these, KOA is the most prevalent and burdensome disorder (1–3).

KOA is a degenerative joint disorder affecting 263 million people worldwide that is responsible for severe pain, joint stiffness, and impaired joint mobility (2, 4). Due to the physical nuisance caused by KOA, patients often complain about a deteriorated quality of life. Besides intense pain, KOA is accompanied by serious economic implications, accounting for billions of dollars of expenses for the treatment and mitigation of the disease consequences (4). KOA is an untreatable condition that can be resolved only by total knee replacement for severe cases (5). Nevertheless, it is feasible to mitigate disease complications by introducing various surgical and non-surgical procedures. Surgical methods involve arthroscopy, osteotomy, and knee arthroplasty (4).

Among non-surgical interventions, exercise interventions, including strengthening training, balance training, aerobic, and neuromuscular exercises, are considered a promising approach for alleviating knee KOA symptoms. In comparison with surgical and pharmacological methods that may pose a certain risk to patients, exercise protocols are more advantageous in terms of safety (1).

Clinical diagnoses and trials of KOA typically evaluate pain, physical function, health-related quality of life (QoL), and, less consistently, biomechanical outcomes and adverse events. Earlier syntheses (for example, Kraus et al. and Goh et al., 2019) (6, 7) concluded that physical activity and structured exercise reduce pain and improve function in people with knee OA, but they also highlighted heterogeneity in exercise types, doses, and follow-up times and noted that benefits often attenuate over months. Many further systematic reviews and meta-analyses (8) have expanded the evidence base, compared exercise modalities (strengthening, aerobic, mind-body, neuromuscular, aquatic, isometric, mixed programs), and investigated different outcome domains. Recent evidence from a comparative network meta-analysis indicates that aerobic exercise may offer the greatest improvements in pain, physical function, gait performance, and quality of life compared with other exercise types, with moderate certainty of evidence (9). However, despite these advances, the overall certainty and consistency of the evidence for pain outcomes across systematic reviews has not yet been comprehensively evaluated, particularly in relation to mechanistic and biomechanical outcomes.

Therefore, it is important to synthesize the latest high-quality evidence on the impact of exercise interventions on pain-related outcomes in patients with KOA to design efficient treatment protocols and methods that prevent symptoms progression. This umbrella review aims to systematically identify, appraise, and synthesize evidence from existing systematic reviews and meta-analyses to determine the effectiveness of therapeutic exercise interventions for adults with knee osteoarthritis, specifically evaluating their impact on pain, physical function, quality of life, and knee biomechanical outcomes. Applying the PICO framework, the review focuses on adults with knee osteoarthritis (Population), evaluates therapeutic exercise interventions (Intervention) in comparison with no intervention, usual care, or other non-exercise controls (Comparator), and examines pain as the primary outcome, with physical function, health-related quality of life, and knee biomechanical function as secondary outcomes (Outcomes).

## 2 Materials and methods

### 2.1 Search strategy

The protocol of the study is registered and available in the Open Science Framework registry (https://osf.io/pqgrv/overview, registration doi: 10.17605/OSF.IO/PQGRV). This umbrella review was performed in accordance with the PRISMA statement. We followed the PRISMA (Preferred Reporting Items for Systematic Reviews and Meta-Analyses) guidelines (10) throughout this umbrella review, supplemented by recommendations for conducting systematic reviews of systematic reviews. The protocol was registered in Open Science Framework.

To conduct a comprehensive systematic search, we used a predesigned search strategy. The systematic search was conducted in the PubMed, Scopus, and Cochrane Library databases from inception to January 15, 2024. We limited the search to articles published in English.

The search terms included:

- For PubMed:

(Osteoarthritis, Knee[Mesh] OR Knee Joint[Mesh] OR “knee osteoarthritis”[tiab] OR “knee OA”[tiab] OR “knee pain”[tiab] OR “knee joint pain”[tiab])

AND

(Exercise Therapy[Mesh] OR Physical Therapy Modalities[Mesh] OR exercise[tiab] OR “exercise therapy”[tiab] OR “physical activity”[tiab] OR physiotherapy[tiab] OR “physical therap*”[tiab] OR rehabilitat*[tiab]).

- For Scopus:

TITLE-ABS-KEY (“knee osteoarthritis” OR “knee OA” OR “knee pain” OR “knee joint pain” OR (knee AND (osteoarthr* OR pain OR “joint pain”)))

AND

(“exercise therapy” OR exercise* OR “physical activity” OR physiotherap* OR “physical therap*” OR rehabilitat*).

- For Cochrane Library:

(MeSH descriptor: Osteoarthritis, Knee, explode all trees OR knee NEAR/3 (osteoarthr* OR “joint pain” OR pain):ti,ab,kw)

AND

(MeSH descriptor: Exercise Therapy, explode all trees OR exercise* OR “exercise therapy” OR “physical activity” OR physiotherap* OR “physical therap*” OR rehabilitat*:ti,ab,kw).

### 2.2 Inclusion and exclusion criteria

The inclusion criteria were as follows:

1. Population: patients with KOA;
2. Intervention: therapeutic exercises;
3. Comparator: medical, surgical methods of treatment, no exercise or therapeutic exercises other than in the interventional group.
4. Outcomes: primary outcome: pain; secondary outcomes: quality of life, functional outcomes, effect on knee biomechanics;
5. Study design: systematic reviews and/or meta-analysis published in English.

Publications were excluded based on the following criteria:

1. Ineligible publication types: narrative reviews, expert opinions, scoping reviews without transparent methods, editorials, commentaries, conference abstracts, and review protocols without completed analyses. These sources do not meet the methodological standards required for umbrella reviews because they often lack reproducibility or comprehensive search processes;
2. Non-relevant diagnosis: reviews focused on knee pathology not corresponding to degenerative osteoarthritis such as inflammatory conditions (rheumatoid arthritis, psoriatic arthritis and others), traumatic knee injuries [Anterior Cruciate Ligament (ACL) tears, meniscal injuries, fractures, and postoperative rehabilitation];
3. Non-relevant populations: pediatric or adolescent cohorts, because their joint structures, disease etiologies, and treatment responses differ significantly from adults. To avoid misclassification bias mixed-population reviews were excluded;
4. Non-relevant interventions: pharmacological interventions, surgical procedures, passive physiotherapy, educational or behavioral interventions;
5. Methodological quality: systematic reviews and meta-analyses were excluded only if outcome data were incomplete or non-extractable despite attempts to contact the authors. This constituted as the minimum quality threshold for inclusion. AMSTAR-2 ratings (low or critically low confidence) were not used as exclusion criteria;
6. Duplicates: in the case when both a preprint and a peer-reviewed article existed, only the final published version was included;
7. articles published not in English: the translation of non-English reviews is not feasible within the resources of the project, and may introduce interpretive error.

### 2.3 Literature screening and data extraction

Two reviewers were involved in the literature screening, Dmitriy Viderman and Akerke Mazhibiyeva. After reviewing the title and the abstract, the full-text articles were retrieved for further evaluation. Literature screening was independently conducted based on the inclusion and exclusion criteria. Any disagreements were resolved through discussion with a third reviewer, Alua Shagirova, until a consensus was reached. Data extraction was conducted by two reviewers, and another reviewer verified the data. We extracted the following data into a descriptive table: author, country, year, citation, goals of the study, types and modes of exercise (cardio, flexibility, resistance), the number of studies included in each systematic review, the number of patients included in the systematic review, the mechanism of action of exercise, complications of therapeutic exercises, advantages/disadvantages of these therapeutic exercises, and study conclusions/comments. Interrater reliability was calculated using Cohen's kappa coefficient (k) (11). The formula applied is as follows:

k=[Pr(a)–Pr(e)]/[1–Pr(e)]

Pr(a) stands for actual observed agreement and it equals:

(a+d)/N

Pr(e) stands for chance agreement and it equals:

$$[(a\;+\;b)(a\;+\;c)\;+\;(c\;+\;d)(b\;+\;d)]\;/\;N^{2}$$

In these formulas a stands for total of how many studies both reviewers agreed to include, b stands for how many studies first reviewer included but the second reviewer excluded, c stands for how many studies first reviewer excluded but the second reviewer included, d stands for how many studies both reviewers agreed to exclude, N stands for total sum of a, b, c, and d.

### 2.4 Quality assessment

Two reviewers independently assessed the methodological quality of each systematic review using the AMSTAR 2 tool, which evaluates 16 items sequentially. Items 2, 4, 7, 9, 11, 13, and 15 are considered critical domains. High quality is attributed to studies with no more than one non-critical weakness. Moderate quality is assigned to those with multiple non-critical weaknesses. Studies with one critical flaw, regardless of other non-critical weaknesses, are deemed low quality. Critically low quality applies to studies with more than one critical flaw, with or without additional non-critical weaknesses. Any disagreements were resolved by discussion with a third reviewer until a consensus was reached.

### 2.5 Data synthesis

Due to heterogeneity among included systematic reviews and inconsistent reporting of comparable effect sizes, no quantitative synthesis was conducted. Publication bias and small-study effects were not assessed through quantitative tests because effect sizes were not consistently reported across reviews. Findings were synthesized narratively. The outcomes were grouped based on reported outcomes and types of therapy. To summarize quantitative findings and methodological the narrative synthesis was supported by structured tables. Figures were used to visually present the key findings.

## 3 Results

### 3.1 Included studies

**Figure 1:**
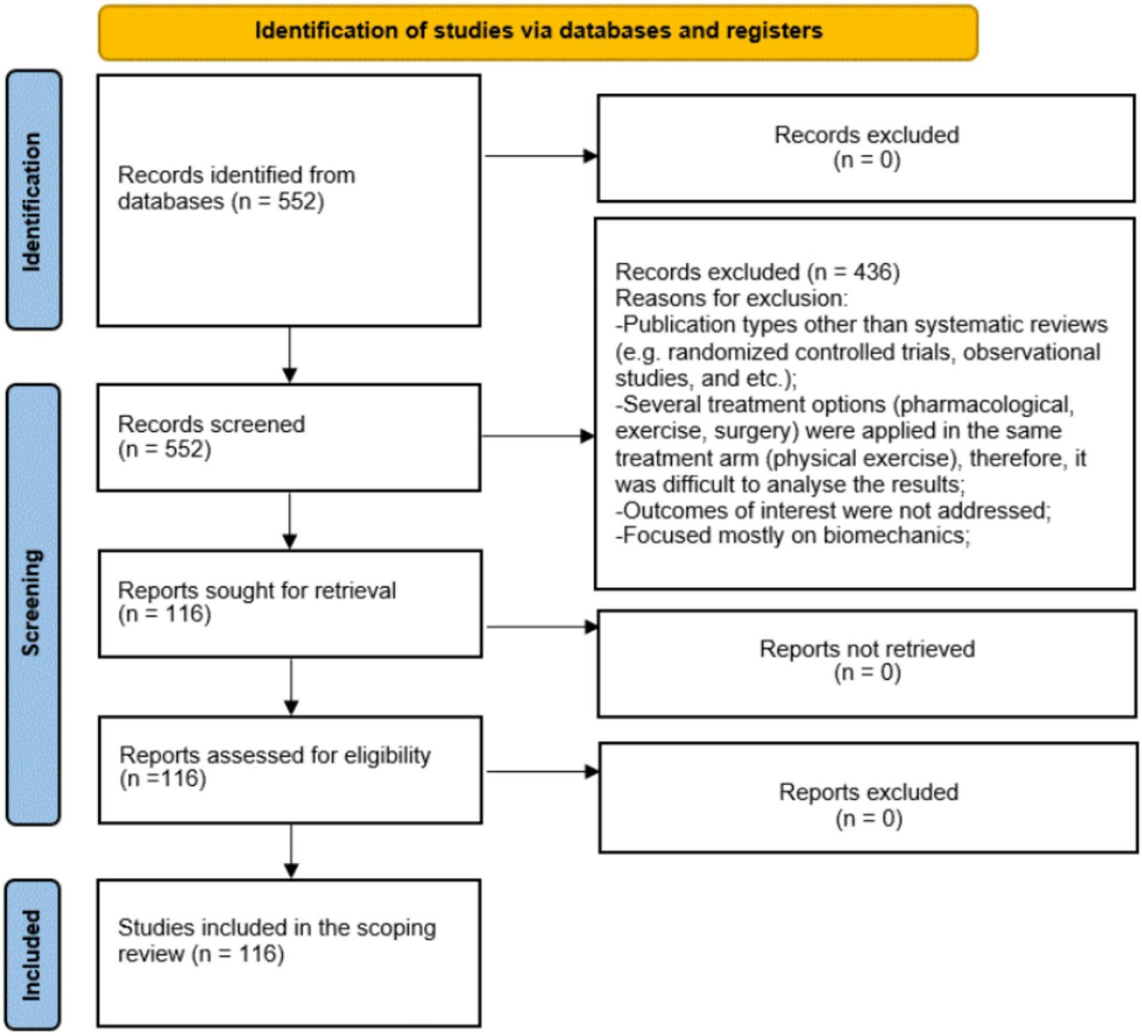
PRISMA diagram. The flow chart shows the process of selecting studies.

We initially identified 552 articles, of which 436 were excluded. A total of 116 systematic reviews and meta-analyses (SRs and MAs) were included in this umbrella review (Figure 1) with the description of reasons for exclusion of irrelevant studies (1–8, 12–119). Study characteristics are described in Supplementary Table S1. Kappa (k) calculation was performed as follows: a was equal 90, b was 20, c was 20, and d was 420. Based on these numbers, observed agreement was found to be 0.924 and expected agreement was 0.679. Based on Pr(a) and Pr(e) kappa was calculated to be 0.76 which shows substantial agreement of interrater reliability.

Based on the AMSTAR-2 assessment, 47 studies were of critically low quality, 47 studies were of low quality, 12 were of moderate quality, and only 10 studies were of high quality. The full assessment is presented in Supplementary Table S2. Most commonly, the included studies failed to satisfy Items 4, 7, 10, 15, and 16. Most studies did not provide a list of excluded studies (Item 4), and few conducted an adequate duplicate study selection and data extraction (Item 7). Likewise, many reviews failed to perform or report an assessment of publication bias (Item 10) and rarely discussed the potential impact of study quality on review findings (Item 15). Reporting of conflicts of interest for both the review and included studies (Item 16) was also frequently absent. These recurring methodological omissions contribute to the predominance of low and critically low (81%) AMSTAR ratings in the evidence base.

### 3.2 Year of publication

**Figure 2:**
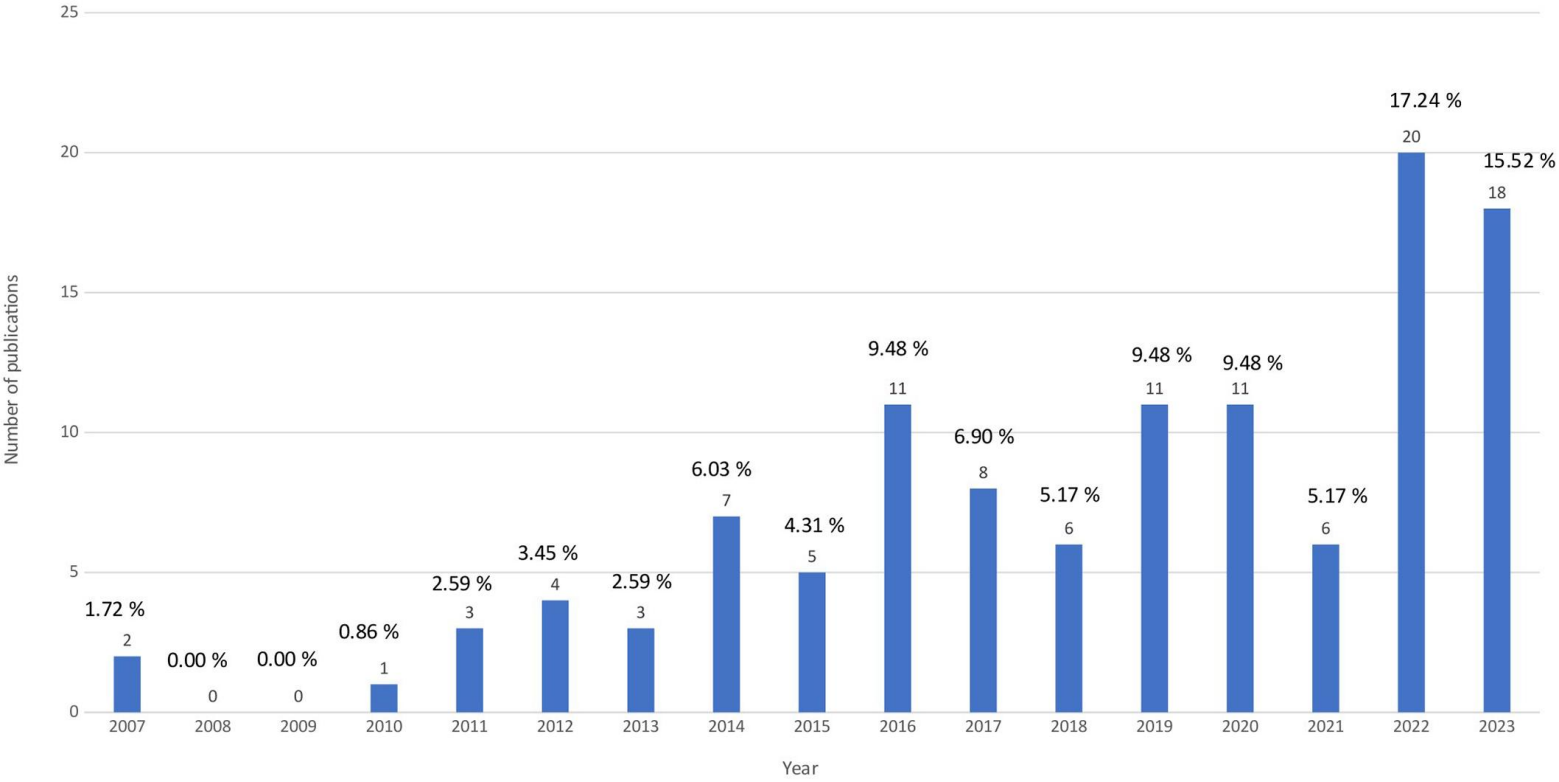
Year of publication. The bar chart shows the distribution of the included systematic reviews by the year of publication.

Overall, the included studies are relatively recent, with the first published in 2007. The majority of the SRs and Mas (96 articles) were published in the last ten years, after 2014 (Figure 2). This may indicate a growing research interest in this field. Such an upward trend suggests increasing recognition of the topic's clinical and scientific relevance. Thus, the available recent evidence expands the evidence on the effectiveness of interventions.

### 3.3 KOA types included in the umbrella review

Knee pain–related conditions included in this umbrella review primarily encompassed knee osteoarthritis, with some reviews also examining combined knee and/or hip osteoarthritis. Most of the articles focused on knee osteoarthritis (KOA), with 83 (71.55%) articles concentrating on KOA (3–5, 8, 12–14, 17, 19–22, 25–30, 32, 34–38, 40, 41, 43–56, 59–63, 65–72, 76, 77, 79–82, 84–89, 91, 93, 96–104, 106, 108–110, 112–115). Knee and/or hip osteoarthritis was examined in 22 systematic reviews (6, 7, 16, 23, 24, 39, 42, 57, 58, 64, 73, 75, 78, 83, 90, 92, 94, 107, 111, 116, 117, 119), constituting 18.97% of the included studies.

### 3.4 Therapeutic exercises used for KOA

Therapeutic exercises play a pivotal role in the management of KOA. It helps mitigate symptoms, enhance joint function, and improve the quality of life. The effectiveness of various types of exercise, including traditional aerobic exercise, strength and resistance training, and flexibility routines, has been consistent throughout the clinical studies. Moreover, traditional Chinese exercises such as Tai Chi and Qigong offer additional benefits, emphasizing balance, mental well-being, and systemic health.

**Figure 3:**
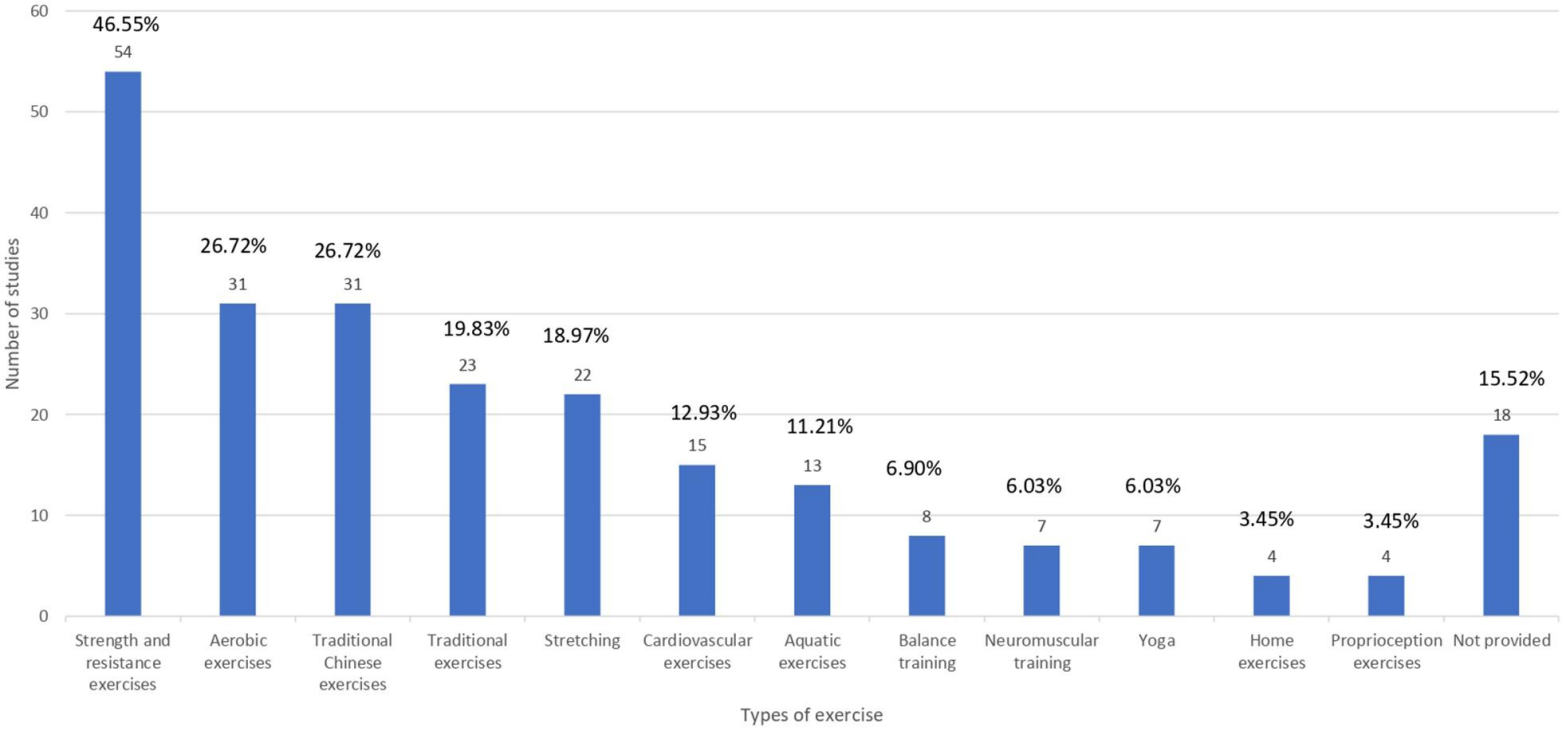
Types of exercises. The bar chart demonstrates the distribution of the included studies by exercise types.

Figure 3 categorizes exercise types. Traditional Chinese exercises—defined as mind–body movement practices originating from Chinese medicine and philosophy, such as Tai Chi, Qigong, Baduanjin, Yijinjing, and related structured routines— were included in 31 articles (3–8, 12–14, 24, 25, 41, 44, 47, 51, 53, 55, 58, 63, 64, 78, 81, 82, 97, 98, 102, 109, 110, 113, 116, 117), making up 26.72% of the 116 articles. The included articles identified cycling, walking, and exercises like squats, step-ups, etc. as traditional exercises. Traditional exercises—defined as conventional, non–mind–body physical training methods commonly used in musculoskeletal rehabilitation, such as cycling, walking, resistance training, strengthening, and stretching exercises (3, 8, 17, 18, 26, 31, 32, 34–36, 38, 43, 44, 51, 60, 78, 97, 104, 107, 109, 111, 116, 117)— made up 19.83% (23 articles) of all the included SRs. Meanwhile, aerobic exercise was examined in 31 articles, making up 26.72%. The most frequent category of exercise was the Strength and Resistance Exercise. It was mentioned in 54 articles (1, 2, 4–7, 13, 20, 22, 24, 27, 39, 40, 45, 48, 49, 51, 52, 56–59, 63–65, 67–69, 71, 77, 79, 83, 84, 86, 88, 90, 95–101, 103, 106, 108–110, 112, 113, 116–119), which is 46.55%.

### 3.5 Types of therapy outcomes in the included studies

**Figure 4:**
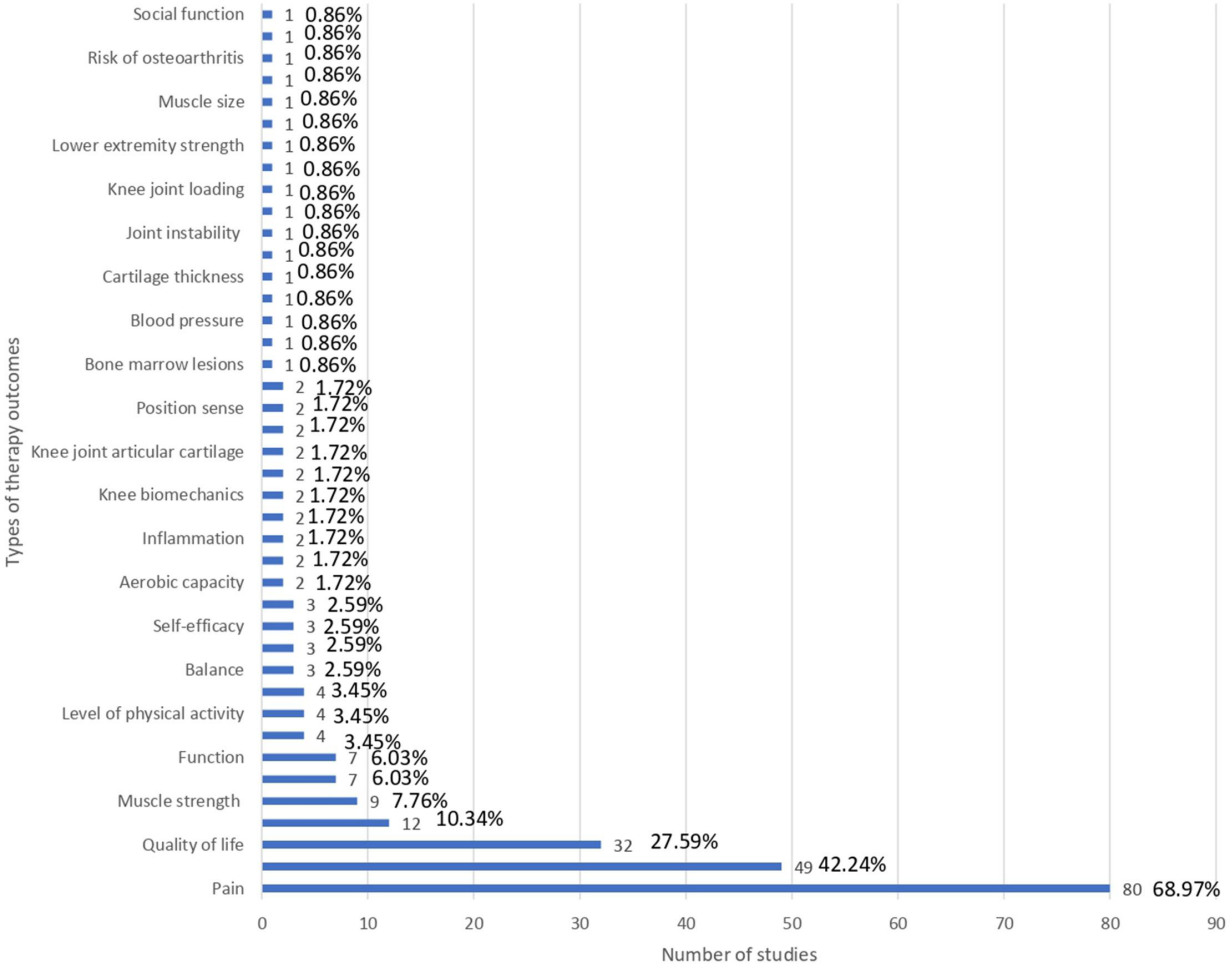
Types of therapy outcomes. The bar chart shows the distribution of studies by therapy outcomes.

Different types of therapy outcomes were reported in the included studies (Figure 4). The top three mentioned outcomes include pain, physical function, and quality of life. Pain was reported as an outcome in 80 studies (68.97%), physical function was reported in 49 (42.24%) SRs, and quality of life was reported in 32 (27.59%) SRs. Several secondary outcomes were also captured, including stiffness, muscle strength, mental well-being, and general function, though each appeared in fewer than 10% of the reviews. A range of more specific or biomechanical outcomes—such as knee range of motion, physical activity levels, walking performance, balance, knee adduction moment, self-efficacy, and others—were reported infrequently, typically in only two to four studies. The least commonly reported outcomes, each appearing in a single review, included physiological, biomechanical, and structural measures such as bone marrow lesions, cartilage thickness, joint instability, sleep, systemic inflammation, and similar parameters. In summary, reporting was heavily concentrated on pain, function, and quality-of-life measures, while more specialized clinical or biomechanical outcomes were only sparsely represented.

### 3.6 Pain outcomes

**Figure 5:**
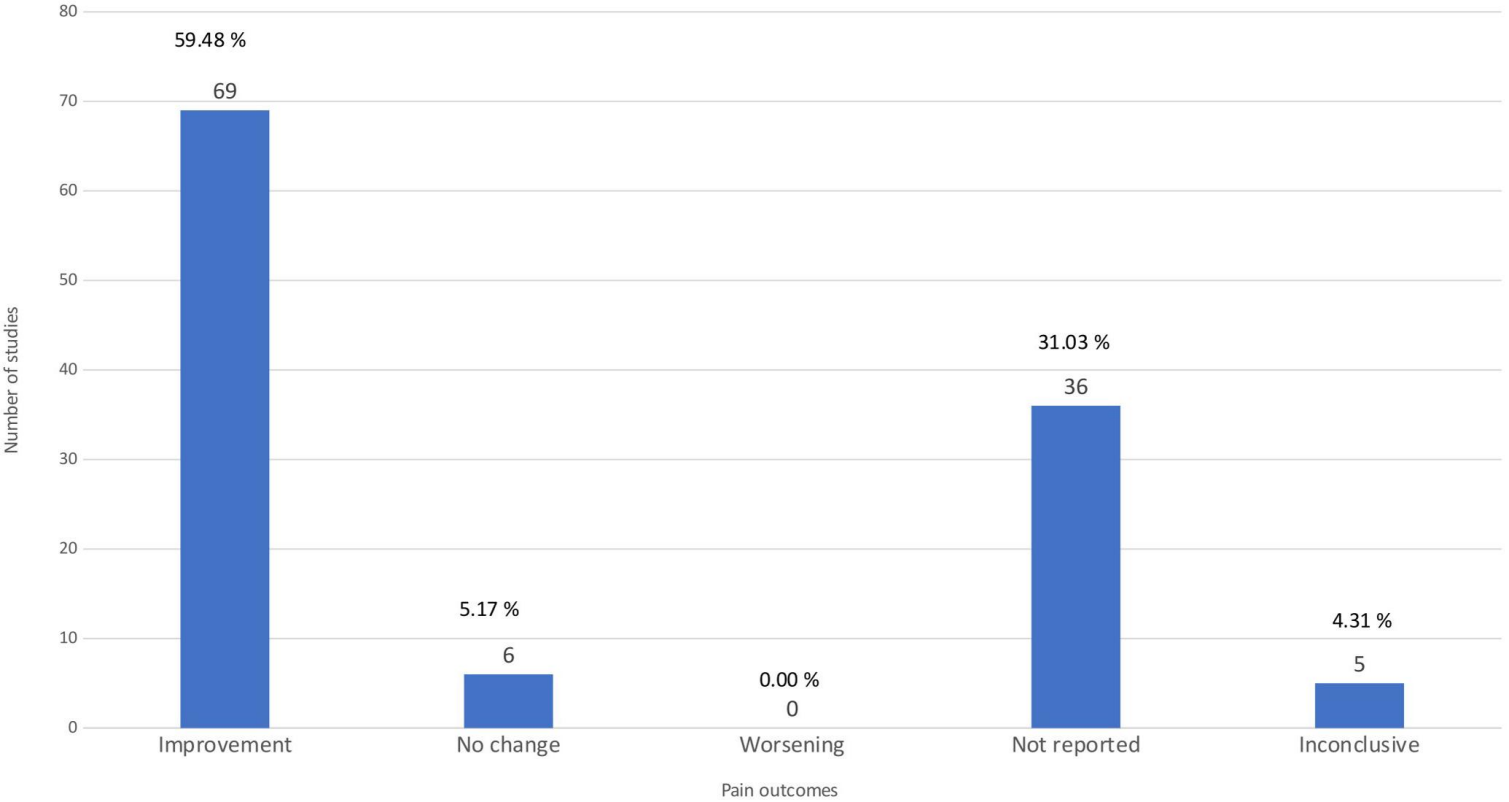
Pain outcomes. The bar chart ilustrates the distribution of the studies by the results of pain outcomes.

In the systematic reviews and meta-analyses concerning KOA, a significant emphasis was placed on the role of physical exercise in managing pain symptoms. As evident from the bar chart (Figure 5), the majority of the studies reviewed, specifically 69, observed an improvement in pain outcomes following exercise interventions (Supplementary Table S3). These interventions encompass a range of exercise types from aquatic to land-based forms, such as Tai Chi and resistance training. Notably, these forms of exercise have been shown to significantly enhance joint function and alleviate pain symptoms (25, 41, 47, 53, 55, 64, 78, 81, 82, 102). Additionally, incorporating behavior change techniques with exercise has also slightly mitigated knee pain, positioning it as a viable non-surgical option for KOA and chronic knee pain management (37, 38, 44, 55). However, several studies presented inconclusive results regarding pain outcomes in KOA and chronic knee pain. This ambiguity highlights the complex nature of KOA and its treatment, where various interventions may yield inconsistent effects on pain symptoms (12, 29, 92, 94, 118). Regarding the evidence quality, among the 69 articles that reported a positive effect on pain, 28 were rated as low quality, 26 as critically low, 10 as moderate, and only 5 as high quality (Supplementary Table S3). Such a distribution indicates that the evidence is largely derived from studies with important methodological limitations. Furthermore, in several studies on KOA, pain outcomes were not reported (3, 4, 15, 16, 21, 23, 26, 30, 31, 34, 36, 42, 45, 47, 52, 59, 60, 69–72, 75, 77, 83–86, 97, 104, 107, 111, 114, 116, 117). This omission highlights a notable gap in KOA research, particularly where the primary focus may have been on other clinical measures such as biomechanical parameters, physical function, or quality of life (QoL).

Finally, a few studies specifically reported no change in pain outcomes following various interventions (35, 49, 62, 66, 99, 112). This finding again underscores the complex and multifactorial nature of KOA and chronic knee pain, where interventions may improve other areas of health or function without necessarily impacting pain levels. It also suggests that managing KOA pain may require more nuanced or individualized treatment strategies that take into account a diverse range of factors influencing patient symptoms.

### 3.7 Physical function outcome

**Figure 6:**
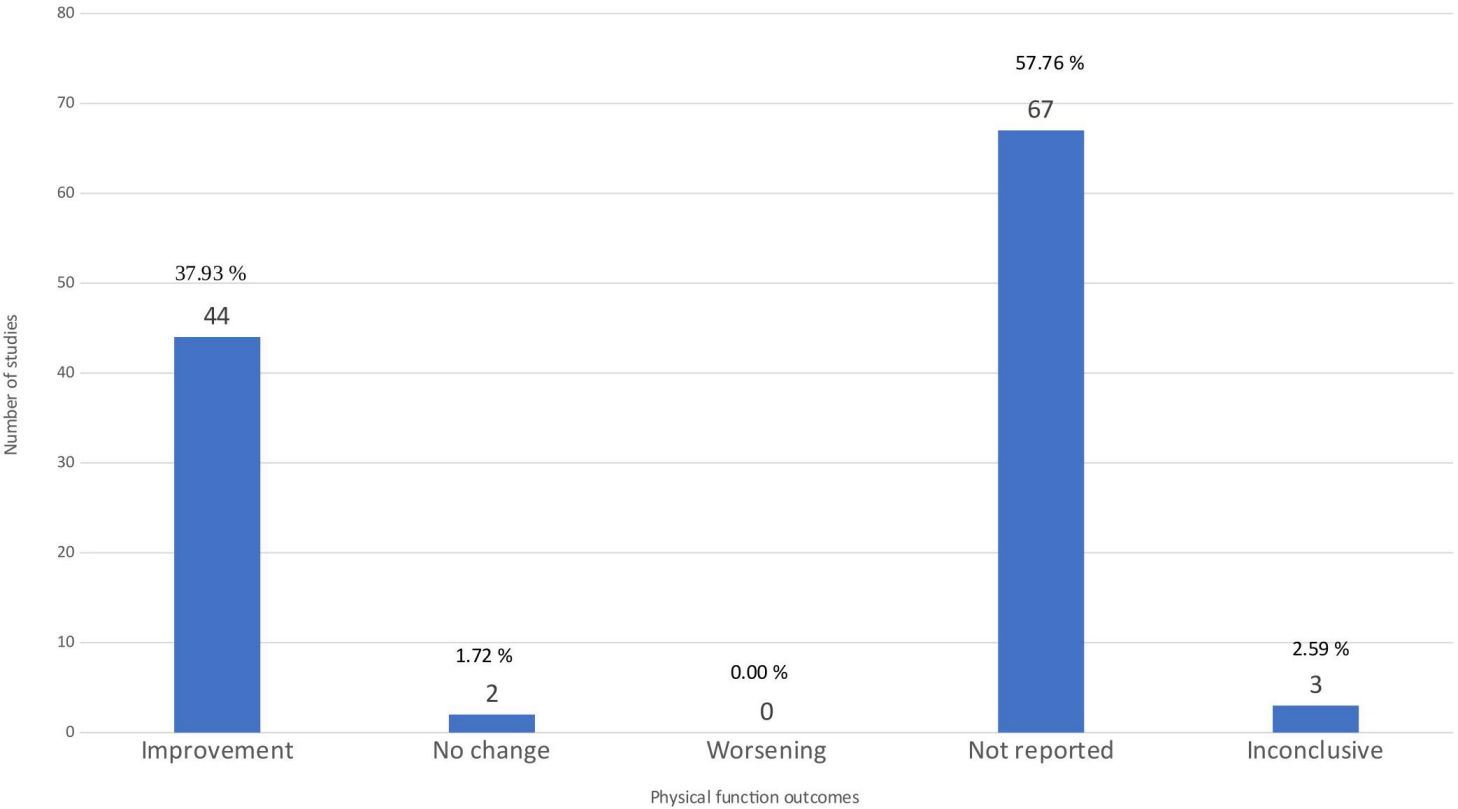
Physical function outcome. The bar chart shows the distribution of studies by the results of the outcome of physical function.

Figure 6 shows that 44 studies (37.61%) reported improvement of physical function after exercise therapy (5–8, 12, 14, 15, 22, 27, 32, 40, 41, 48, 50, 51, 53–58, 64, 66–68, 72–74, 76, 78–82, 88, 89, 91, 94, 95, 99, 102, 105, 112, 115), two studies (1.72%) reported no change (49, 62), three studies (2.59%) reported inconclusive results (29, 92, 111), and none reported worsening. In these studies, physical function was evaluated using different scales, such as The Western Ontario and McMaster Universities Arthritis Index (WOMAC) physical function subscale (46%), Late Life Function and Disability Scale Index (LL-FDI) (33%), and Arthritis Impact Measurement Scales (21%). The remaining 67 articles did not report physical function as a therapy outcome. Among the 44 articles reporting a positive effect on pain, 18 were rated as low quality, 16 as critically low, 6 as moderate, and only 4 as high quality. Three articles that reported inconclusive findings consisted of one moderate-, one low-, and one critically low-quality review. Two articles that reported insignificant effects were of low and critically low quality (Supplementary Table S3). Overall, these distributions indicate that the evidence base supporting positive effects is concentrated in reviews with notable methodological limitations. Therefore, the apparent consistency of positive findings should be interpreted with caution, and confidence in the overall evidence remains restricted.

### 3.8 Quality of life

**Figure 7:**
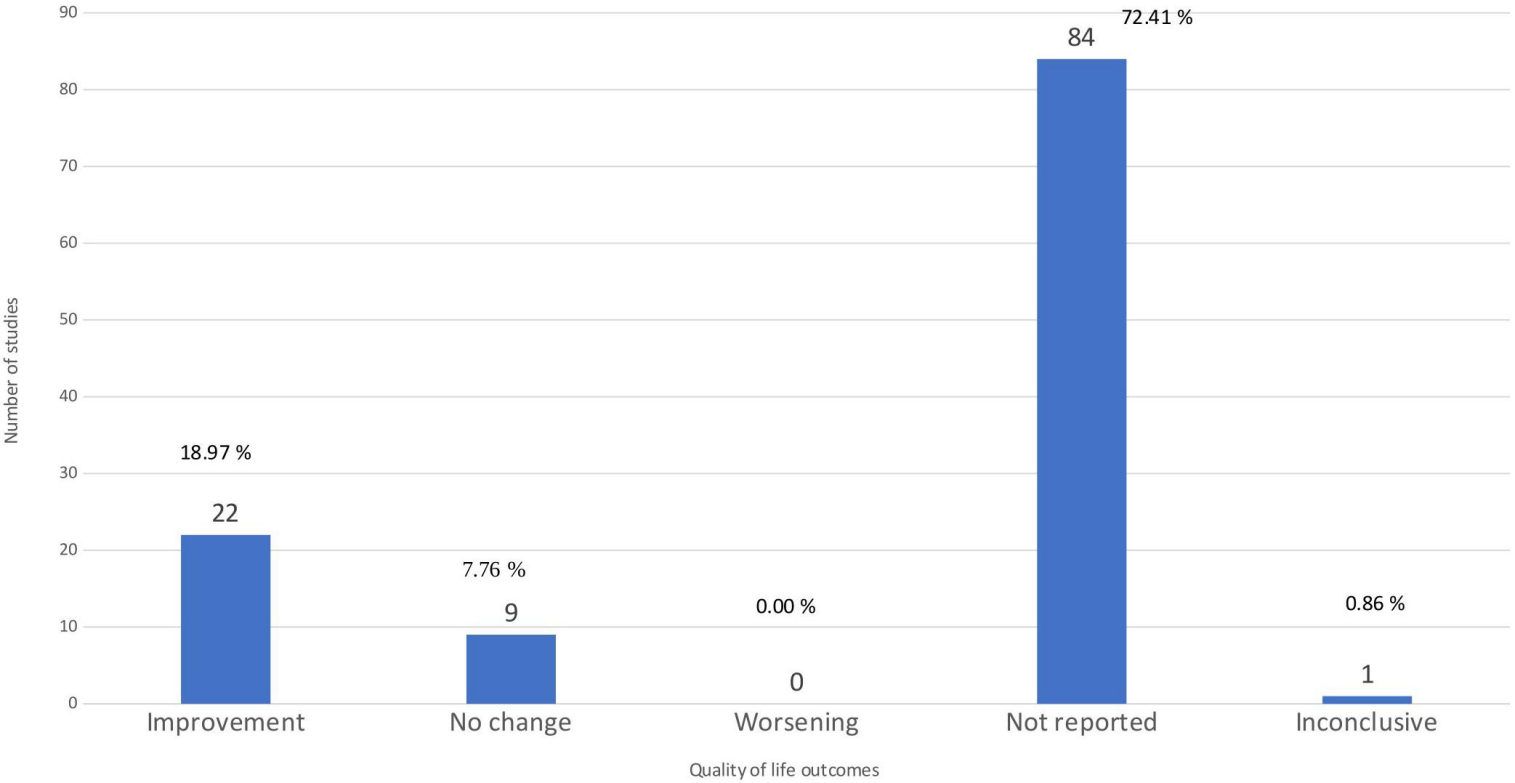
Quality of life outcome. The bar chart demonstrates the distribution of studies by the result of the outcome of quality of life.

Figure 7 shows that 22 studies (18.97%) reported improvements in quality of life (5–8, 32, 48, 50, 51, 55, 58, 60, 64, 72, 79–82, 88, 90, 91, 93, 105), nine studies (7.76%) reported no change (15, 35, 40, 41, 62, 65, 66, 99, 112), one study was inconclusive (29), and none reported worsening. Quality of life was not examined as an outcome of therapy in the prevailing number of studies, namely 84 papers (72.41%). Instead, these studies assessed the quality of life using approaches such as the Arthritis Impact Measurement Scales 2 Short Form (AIMS2-SF) (43%), Assessment of Quality of Life (AQoL) (38%), and Medical Outcomes Study Questionnaire Short Form (SF-36) (19%). Among the 22 articles reporting a positive effect, 11 were rated as low quality, 7 as critically low, 2 as moderate, and 2 as high quality. One article with inconclusive findings was of moderate quality. Among the nine articles reporting insignificant effects, four were rated as low quality, four as critically low, and one as moderate (Supplementary Table S3). In summary, these distributions indicate that much of the available evidence is derived from reviews with substantial methodological limitations.

### 3.9 Knee biomechanics

We combined several outcomes into ‘knee biomechanics’, which reflect the coordination and structure of knee joints. The outcomes were (a) the Knee Adduction Moment (KAM), which measures the loading of the medial tibiofemoral compartment, (b) the Knee Flexion Moment (KFM) and (c) the Knee Compression Force (KCF), representing the bending force applied and the pressure exerted on the knee joint, respectively (1, 17). Most studies, however, did not examine biomechanical variables.

**Figure 8:**
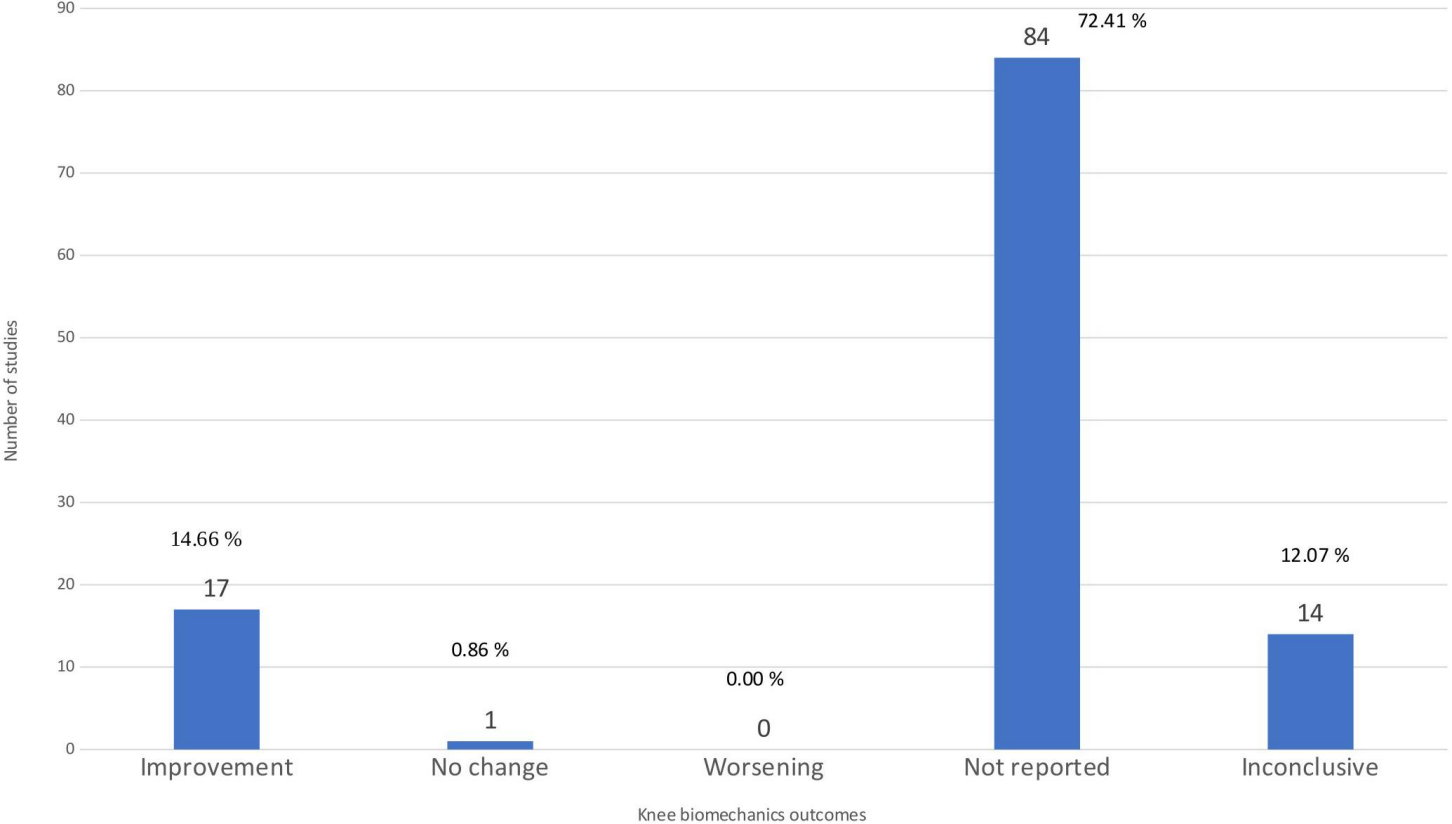
Knee biomechanics outcomes. The bar chart shows the distribution of studies by the results of the outcome of knee biomechanics.

Figure 8 illustrates that 17 articles reported improvements in knee biomechanics, indicating positive effects of physical therapy. However, the majority of the articles (84) did not report specific biomechanic outcomes, and 14 articles obtained inconclusive results, reflecting the ongoing challenge in capturing complex biomechanical changes. Interestingly, only one study reported no change, and none indicated a worsening in biomechanics, which may suggest a trend toward the beneficial impact of exercise therapy in the mechanical aspects of the knee joint (28).

One study focused exclusively on the Knee Adduction Moment, a crucial indicator of load distribution, which is particularly relevant in the context of osteoarthritis (1). However, the findings regarding the changes in KAM were inconclusive, suggesting that improvements in clinical symptoms such as pain and function might not directly correlate with biomechanical changes in the knee. The reviewed studies also evaluated various exercise therapy modalities for knee osteoarthritis, including general strength training, aerobic exercises, and specific physical therapy regimens aimed at improving joint mobility and muscle function (2, 4, 19, 22, 31, 34–36, 44, 48, 52, 54, 68–71, 109). These therapies generally improved clinical outcomes, indirectly indicating the beneficial effects on knee biomechanics by enhancing muscle strength and reducing joint stiffness. Although direct measurements of changes in KAM, KFM, and KCF were less frequent, the broader implications for knee biomechanics through improved muscle strength and stiffness have been well-reported, highlighting the positive outcomes of therapeutic interventions. However, many studies have reported that while several exercise therapies can reduce pain, the conclusions regarding changes in knee biomechanics remain inconclusive (17, 23, 24, 30, 35, 47, 49, 62, 76, 77, 88, 95, 96, 112). Among the 17 articles reporting a positive effect, 5 were rated as low quality, 11 as critically low, and only 1 as high quality. Fourteen articles reported inconclusive findings, of which 7 were critically low, 3 were moderate, and 4 were low quality. Only one article reported an insignificant effect, and it was rated as low quality (Supplementary Table S3). This distribution shows that most evidence originates from reviews with substantial methodological limitations. Thus, the overall confidence in these findings is restricted, and conclusions should be interpreted with caution.

Given the substantial number of studies not reporting specific biomechanical outcomes or reaching inconclusive results, the need for more rigorous randomized controlled trials becomes ever more apparent to solidify our understanding of the advantages of exercise therapy for knee osteoarthritis.

### 3.10 Quality assessment

Figure 4 illustrates that pain, physical function, and quality of life are the most frequently reported outcomes across the included reviews. Knee biomechanics appears as the fourth most common category, although it is presented under varying terminology across studies. Next, Figure 5 further shows that, despite pain being the most frequently reported outcome (68.96%), almost one-third of studies (31.03%) do not report it. Figure 6 demonstrates that physical function is also underreported, with only 57.76% of studies including it. Figure 7 indicates that quality of life is largely absent, with 72.41% of papers not reporting. Likewise, Figure 8 shows that knee biomechanics is inconsistently reported, with 72.41% of studies omitting it.

**Figure 9:**
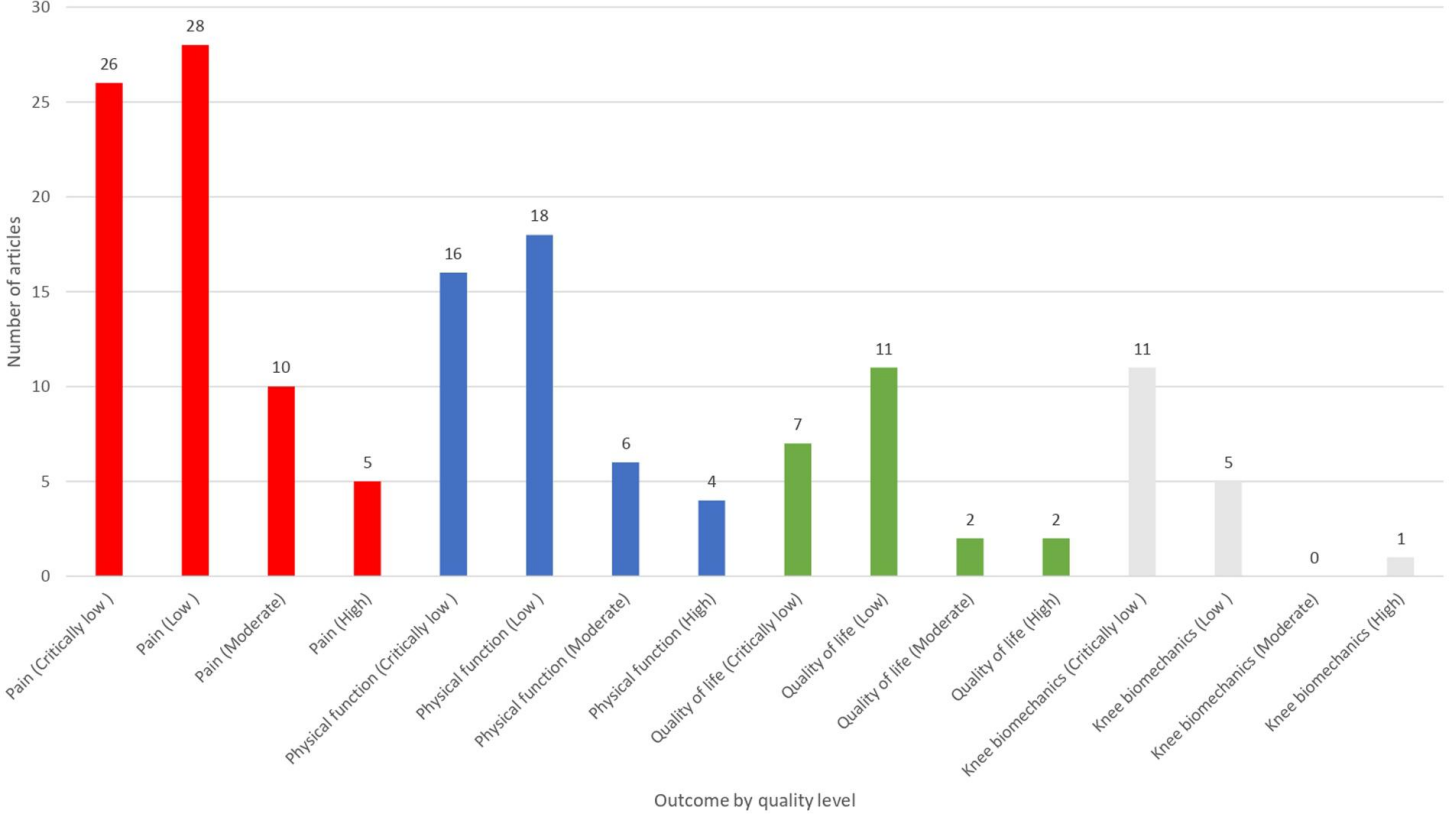
Distribution of methodological quality by reported outcome improvement.

Across Figures 5–8, the outcomes that are reported tend to show predominantly positive findings, which may reflect the tendency of reviews to highlight statistically significant results. AMSTAR-2 revealed that these conclusions are primarily derived from low or critically low-quality evidence, limiting confidence in positive effects. Therefore, Figure 9 was created to depict the distribution of positive findings across the four major categories when stratified by methodological quality. The figure shows that most positive reports originate from low or critically low-quality reviews, although moderate- and high-quality studies are still present, albeit at roughly half the frequency of lower-quality evidence. Together, these patterns underscore the need for cautious interpretation of reported improvements.

## 4 Discussion

In this umbrella review, we analyzed the impact of exercise therapy on outcomes in patients with KOA. The studies comprised various types of exercises including traditional aerobic exercises, strength/resistance training, flexibility exercises, and traditional Chinese practices such as Tai Chi and Qigong. The most common outcomes were pain, physical function, and quality of life. Most studies (59%) reported a reduction in knee pain. Among the studies that assessed the effect of PE on knee function, and among those assessing the effect of PE on the quality of life, the majority reported an improvement. Other outcomes that have been positively affected by physical exercise include stiffness, muscle strength, mental wellbeing, and function. However, substantial knowledge gaps remain. Despite the apparent predominance of positive findings, the AMSTAR-2 appraisal revealed that the majority of evidence underpinning these conclusions originates from reviews of low or critically low methodological quality. Common deficiencies include inadequate reporting of excluded studies, lack of publication bias assessment, and insufficient consideration of how study quality influences conclusions. As a result, the strength of evidence supporting many reported benefits remains uncertain, particularly for outcomes such as quality of life and knee biomechanics, which are both underreported and inconsistently defined. Furthermore, the uneven reporting of outcomes (with a significant proportion of reviews omitting pain, physical function, or biomechanical measures) limits the comparability of findings and hinders the development of outcome-driven policy recommendations. Importantly, several moderate- and high-quality meta-analyses, such as those by Goh et al. and Kraus et al. (7, 64), provide more robust support for the beneficial role of structured exercise, particularly strength and resistance training, reinforcing the position of exercise therapy as a core non-pharmacological strategy in KOA management.

The available evidence shows that exercise therapy may mitigate symptoms and improve joint function and quality of life. Furthermore, traditional Chinese exercises might offer additional benefits, such as balance, mental well-being, and general health. The evidence, however, is limited, and this question needs further exploration (15). The review provides preliminary support for the short-term effectiveness of aquatic exercise in decreasing pain in people with KOA (22). Moreover, exercise combined with educational sessions significantly increased physical activity and reduced pain in KOA patients (24).

Due to exercise's capacity to effectively alleviate pain and improve physical function and quality of life, healthcare professionals should be encouraged to include various exercise programs in the treatment of patients with KOA. Both supervised and unsupervised exercise types are effective, and the decision to practice either should be based on the resources, capabilities, and motivation of the patient. There are other considerations to keep in mind. Practices such as Tai-chi and Qigong include cultural aspects of the Chinese tradition. Therefore, to maximize their effectiveness, such practices should be made accessible to other cultures. Moreover, socioeconomic status may affect a person's access to specific exercise types by, for example, restricting access to the required equipment and facilities. In addition, the possibility to engage in certain exercise forms could be influenced by whether the person lives in an urban or rural area, and by the general safety of performing them. Therefore, exercise programs should ideally be individualized. This would also allow for higher fidelity to the program.

The available evidence (1–8, 12–119) shows that a wide variety of therapeutic exercises including strength, aerobic, traditional Chinese exercises (Tai Chi, Qigong) and others may beneficially affect the outcomes in patients with KOA. The decision, which specific type of exercises to include in the program should depend on individual patient characteristics and preferences, such as age, pain severity, and severity of joint damage. In fact, it is important to maintain the key qualities of musculoskeletal system, such as strength, endurance, flexibility, mobility, and balance. Another important goal is to focus not just on knee pain but on the entire patient living with KOA. Therefore, it is important to maintain cardiovascular, respiratory, nervous, musculoskeletal system as whole and that can be achieved only by training through different exercises listed above. Therapeutic exercise should be attempted before surgical treatment, which should be considered if therapeutic exercise therapy has failed.

On a larger scale, policies should be implemented to support the creation and funding of community programs to deliver exercises that are accessible to individuals with KOA. Policymakers should promote a continuous education and training program for healthcare providers to include the most recent evidence-based exercise interventions for KOA. Public health campaigns could raise awareness on the overall benefits of exercise, especially in people with KOA. This would facilitate early intervention and prevention of long-term consequences. These policy recommendations are primarily informed by findings from the subset of systematic reviews with moderate or high methodological confidence, which consistently reported beneficial effects of exercise particularly for pain reduction and functional improvement. It is also important to further investigate the issue as certain questions remain open. Thus, there is a diversity in physiology among people with different ethnic and genetic backgrounds.

Additionally, it is important to consider the types of studies that would be most beneficial for further research. Firstly, randomized controlled trials should be conducted to compare different exercise therapies and their combinations. Secondly, longitudinal studies should be performed to analyze the progression of KOA over time. In summary, although existing evidence supports the potential effectiveness of exercise therapy for KOA (particularly for pain reduction) policy recommendations should be formulated with caution.

### 4.1 Limitations

Methodological limitations: A COSMIN-based evaluation (COnsensus-based Standards for the selection of health Measurement INstruments) of measurement properties was not conducted because the included reviews provided insufficient and inconsistent reporting on the validity, reliability, and responsiveness of the outcome instruments. Publication bias or small-study effects were not feasible due to heterogeneous reporting of effect sizes across the included reviews. No formal assessment of overlap such as Corrected Covered Area and citation matrix among the included systematic reviews was conducted. This could have led to data duplication and an artificial inflation of the apparent strength and consistency of the evidence. Another limitation is that the search included English-only articles which potentially limits the comprehensiveness of the evidence base. Finally, a key limitation was the lack of standardized measurement of knee biomechanics across included reviews, limiting comparability and synthesis of biomechanical outcomes.

## 5 Conclusions

Exercise-based interventions are associated with improvements in pain and physical function in knee osteoarthritis. The evidence for quality of life and knee biomechanics is less consistent and less frequently reported. Although positive effects are commonly observed, the overall certainty of evidence is limited. A smaller number of moderate- and high-quality reviews support these findings, but they constitute a minority of the evidence base. Thus, exercise therapy should remain a key component of non-pharmacological management, yet current conclusions should be interpreted with caution.

## Data Availability

The original contributions presented in the study are included in the article/Supplementary Material, further inquiries can be directed to the corresponding author.

## Supplementary material

The Supplementary Materialfor this article can be found online at: https://www.frontiersin.org/articles/10.3389/fpain.2026.1717540/full#supplementary-material
