# Supplementary material for: Impact of therapeutic exercises on pain-related outcomes in patients with knee osteoarthritis: an umbrella review of 116 systematic reviews
Source: Front Pain Res (Lausanne). 2026 Mar 10;7:1717540. doi: 10.3389/fpain.2026.1717540 (PMC13008949; doi:10.3389/fpain.2026.1717540)
Supplement: Supplementary file 1 [file Supplementaryfile1.docx]

Supplementary Material

**Table 1.** Study characteristics.

|  | **Author, country, year** | **Types and modes of exercise** | **N of studies; N of participants** | **Advantages/disadvantages** |
| --- | --- | --- | --- | --- |
| 1 | Cottmeyer 2023 | Strength training and exercises | 9; 1019 | Advantages: Improving joint function, reducing pain, increasing the level of physical activity, relatively safe, and has no significant side effects.  Disadvantages: A limited number of positive results, exercises are not suitable for all people (not suitable for people with serious injuries), the need for constant monitoring and consultation |
| 2 | Lopes 2023 | Strengthening exercise, exercise therapy | NG; 3067/529/30/148/3963 | Advantages: Comprehensive analysis, detailed statistical analysis, Improvement of knee biomechanics, pain reduction  Disadvantages: Limited number of studies, research bias |
| 3 | Massey 2022 | Walking, stretching, tai chi | 72; 9492 | Advantages: Convenience, accessibility, saving time and resources (they do not require a lot of time and resources, which makes them more attractive to patients)  Disadvantages: Low level of commitment, less effective results |
| 4 | Sheikhhoseini 2023 | Tai Chi, Baduanjin Qigong, proprioceptive exercise, pilates, resistance, strength training exercises, sensorimotor training | 17; 847 | This study does not mention the advantages or disadvantages of any particular method. |
| 5 | Si 2023 | Strengthening exercises + kinesthesia exercises (84%), balance and agility, tai chi (8%) | 12; 1442 | Advantages: Physical exercises at home help to reduce pain and improve physical functions and quality of life in people with osteoarthritis of the knee joint.  Disadvantages: Not specified |
| 6 | Zhang 2023 | Traditional Chinese Exercises | 17; 1174 | The study does not provide a direct comparison of the advantages and disadvantages of TCEs compared to other treatment methods. |
| 7 | Puts 2023 | Aerobic exercise (e.g., Walking, cycling, swimming), strength training, flexibility training, and Tai Chi | 21; 1374 | Only a few of the studies included in the study examined the immediate effects of exercise; the majority were cytokine-focused. Additionally, these studies used a wide range of research methodologies. More high-quality research, ideally randomized controlled trials (RCTs), spanning a wide range of these indicators, is required to address these limitations and gain a better understanding of the potential links between short- and long-term exercise effects on inflammatory markers and BDNF. |
| 8 | Chang 2023 | Chinese exercise (TCE), which includes Tai Chi, Baduanjin, Yijinjing, and Wuqinxi | 17; 1122 | The study did not specifically mention any advantages or disadvantages of traditional Chinese exercise for patients with knee osteoarthritis. It only reports the effectiveness of the exercise in improving pain, stiffness, physical function, and mental health scores compared to the control group. |
| 9 | Mo 2023 | Aquatic exercise, resistance training, Traditional exercise, Stationary cycling, Yoga, Tai chi | 39; 2646 | Advantages: Exercise therapy has been proven to considerably reduce joint pain, enhance bodily function, and enhance patients' quality of life. Exercise therapy is a non-surgical strategy that uses the patient's own strength, the support of the therapist, or rehabilitation tools to improve the functional state of any part of the human body.  Disadvantages: The study also points out that not all people may benefit from exercise therapy, and some may need alternative types of care like medication or surgery. Furthermore, because participants and researchers were not blinded in some of the systematic reviews' studies, there was a high chance of bias, which could have an impact on the validity of the findings. |
| 10 | Chaudhry 2023 | Unsupervised home exercise | 11; 1008 | *Unsupervised home exercises*  Advantages: Convenience & cost reduction: people who can't move around can exercise without leaving home  Equivalent advantages: home exercises can provide the same results as classes under the supervision of specialists.  Disadvantages: Without supervision, the patient may perform the exercises incorrectly, and this may slow down the recovery process.  *Official therapy programs under supervision*  Advantages: Specialized care: individually programmed and attentive medical intervention. Instant feedback and encouraging compliance with the regime  Disadvantages: Inconvenience for people with limited mobility and limited access |
| 11 | Clausen 2023 | The review does not provide an exhaustive list of the forms of exercise investigated. | 8; 101 | The review does not provide a specific method or treatment to evaluate its advantages or disadvantages. |
| 12 | Yokoyama 2023 | Step-ups, side lowers (with dumbbells) with five degrees of difficulty, bilateral, split, and unilateral squats, side-lying hip abduction, clam, bridging, and knee extension movements, Forward and backward sliding or stepping, side exercises, and many others | 20; 1119 | Advantages: Potential mediators of the elevated initial peak KAM include better periarticular knee pain and muscle strength following exercise therapy. Various combinations of land exercise modalities result in varying degrees of improvement in WOMAC pain and function.  Disadvantages: No review of aquatic exercises was done, which may show a better performance compared to land-based exercises. |
| 13 | Singla 2023 | Preoperative fitness level is significant for rehabilitation. Exercise therapy before surgery might have a distinctive influence on postoperative recovery and subsequent physical activity. | 21; 647 | Advantages: This systematic review and meta-analysis offer a comprehensive, unbiased summary of knee extensor strength after total knee arthroplasty. It minimizes bias and enhances study selection and data extraction transparency.  Disadvantages: Varying study quality may affect estimated validity and reliability. Some studies exhibit high bias risk due to confounding, missing data, and intervention deviations. Meta-analysis can only summarize evidence and can't address individual differences or other influencing factors. |
| 14 | Xu 2023 | The review does not provide much information about the types of exercises. Mostly, it mentions knee-bending physical activities and  Unilateral high-impact exercise | 18; NG | Advantages: Papers make good observations and correlations. The results of this study indicated that both light and vigorous PA might be important for the maintenance of cartilage thickness/volume.  Disadvantages: Papers provide a lack of evidence. Unable to indicate a safe threshold of PA. Some eligible studies included participants with potential structural changes visible on MRI only at baseline. |
| 15 | Malik 2023 | The researchers have conducted a variety of exercises. They included range-of-motion, strengthening, stretching, balance, and cardiovascular exercises done 2 to 5 days a week for 2 to 14 weeks. | 14; 820 | Advantages: Pain relief; Improved range of motion; Muscle strength.  Disadvantages: Cost of treatment; Requires regular sessions |
| 16 | Patterson 2023 | No specific exercises were mentioned | 14; NG | Advantages:  Quantitative results, including cartilage thinning and joint space narrowing, are sensitive and can identify structural changes over shorter follow-up periods.  Clinical outcomes like knee strength may be more strongly correlated with structural outcomes like radiographic OA (an increase in Kellgren-Lawrence grade) and cartilage lesions (an increase in WORMS grade). Structural results are more likely to reflect a significant change in joint disease state.  Disadvantages: Measures a small change in disease status. Less sensitive to change over shorter follow-ups. |
| 17 | Xu 2023 | Aquatic exercises, mostly stretching, strength, and aerobic exercises | 22; 1394 | Advantages: High satisfaction rates. Reduced joint forces in the knee joint. The heating effect of warm water may accelerate blood circulation. The risk of adverse events in land-based exercise was much higher than that in aquatic exercise.  Disadvantages: The need for specialized equipment or facilities. The risk of water-related illnesses or injuries increases if safety precautions and good hygiene are not taken. |
| 18 | Coburn 2022 | No exercises are mentioned, as the article focuses on the effect of running on the knees | 24; 446 | Advantages: Much statistical data is analyzed and presented in tables. The assessment of risk of bias was done.  Disadvantages: Only included research where results for cartilage were obtained before and after running. Small samples of MRI evaluations. |
| 19 | Sasaki 2022 | Any kind of land- or aquatic-based exercise program, including mind-body activities (yoga, tai chi, and qigong), cardiovascular activity, strengthening, and stretching, as well as integrated interventions that use multiple of these exercises | 20; 2350 | Advantages: It was stated that in the long term, the exercises in combination with therapy may improve pain; however, further studies are required. The GRADE framework was applied to assess the data. To evaluate the methodological aspects, the revised Cochrane risk-of-bias tool for randomized studies was employed.  Disadvantages: Ten studies indicated a high risk of bias in at least one component, whereas just one study showed a low risk of bias in all five components. Low GRADE evidence in most assessments. The inclusion criteria for OA severity were not specified in this study, and no subgroup analyses based on OA severity were carried out. |
| 20 | Guo 2022 | Traditional Chinese exercises, Wu Qin Xi exercises, Tai Chi, and qigong | 7; 668 | Advantages: WQX is more effective than other gong techniques, including Tai Chi, in helping middle-aged and elderly people improve their lung function and reduce oxidative stress. WQX is easier to learn as it has only 10 sets of movements compared to Tai Chi.  Disadvantages: There is currently no scientific evidence to support the WQX exercise's safety. It is important to do it under professional supervision. |
| 21 | Calaido 2022 | There were included aquatic exercises, isometric exercises, aerobic training, treadmill walking program, underwater treadmill exercise, weight-bearing, and non-weight-bearing exercises | 10; 534 | Advantages: Improved joint stability; Reduced pain; Reduced overload on the knee joint.  Disadvantages: Overdoing exercises may negatively impact the joints. The absence of published research fulfilling the review's inclusion requirements may have hindered the development of a consensus regarding the benefits and side effects of PE used alone to increase functional capacity in people with KOA. |
| 22 | Guo 2022 | Any land-based exercise program, including muscular strengthening, flexibility, and cardiorespiratory exercises | 15; 1436 | Advantages:  High-compliance groups significantly improved stiffness indicators. Benefit from flexibility exercises. KOA may cause alterations in the patterns of muscle activation, such as a reduction in the activation of the quadriceps, which are utilized to deal with pain and bend the knee.  Disadvantages: The drawback of combining resistance training with aerobic exercise in the same session is the molecular reaction, whereby aerobic exercise raises the muscle's mitochondrial content while resistance training enhances the myofibrillar protein response. |
| 23 | Wu 2022 | Kinesio taping (KT) to improve pain and knee function | 16; 642 | Advantages: A combination of KT and physical activity may alleviate pain for individuals with knee osteoarthritis.  Disadvantages: It had a negative influence on knee function improvement. |
| 24 | Ariie 2022 | Exercises with behavior change techniques (Exercise adherence can be increased by using (bcts), which are "observable, replicable, and irreducible components of an intervention designed to alter or redirect causal processes that regulate behavior.") | 21; 1623 | Advantages: A review revealed that particular BCTs are beneficial for persons with lower limb osteoarthritis.  Disadvantages: Studies could not prove if combining BCTs with exercise increased the risk of adverse outcomes. Exercise with four or more bcts is typically less beneficial for outcomes related to knee pain and mobility. |
| 25 | Silva 2022 | Trunk lean, Toe out, medial knee thrust, medial weight transfer to the foot, Toe in, wider steps. | 17; 362 | Advantages: Toe-in reduced KAM1, Ipsilateral trunk lean reduced KAM1 and reduced KAM impulse, toe-out reduced KAM2.  Disadvantages: Toe-in reduced KAM1 (medium effect) but increased KAM2 (small effect). |
| 26 | Granicher 2022 | Preoperative exercise therapy that may improve patients’ physical status preceding  Orthopedic surgeries. | 16; 968 | Advantages: Before and for up to 3 months after the initial total knee arthroplasty (TKA), engaging in exercise therapy prior to surgery enhances knee function. Prehabilitation enhances or even improves knee functionality and may lessen the chance of function decline.  Disadvantages: Which particular workouts or therapy settings are most helpful is unknown. |
| 27 | Jurado-Castro 2022 | Exercise-based nonpharmacologic/nonsurgical therapies have a direct positive impact on physical function. | 64; 410 | Advantages: Patients with KO and serious comorbidities can benefit from individualized exercise therapy to improve their physical functioning (Weight loss results in a long-lasting decrease in the joint discomfort it causes).  Disadvantages: Despite the health benefits of exercise, certain people may benefit from walking up stairs but not down them, in which case using an elevator would be safer to prevent falls. |
| 28 | Rotini 2022 | Either supervised or unsupervised, home exercises or performed at the gym | 6; NG | Arthroscopic surgery: Better improvement in the pain scores and short-term functional outcomes;  Exercise treatment: A significant advantage over cautious exercise therapy was not provided by arthroscopic surgery.  There were no differences in functional results or pain scores between arthroscopic surgery and exercise therapy at long-term follow-up. |
| 29 | Saueressig 2022 | Surgical and rehabilitation therapy | 3; 0 | Surgical: Meniscal injury demonstrated a long-term, but weakly supported, trend in favor of primary surgery.  Rehabilitation therapy: radiological knee osteoarthritis indicated a tendency that marginally favored predominantly rehabilitative treatment. |
| 30 | Fernandez-Matias 2022 | Arthroscopic partial meniscectomy (APM) plus exercise | 4; 713 | Advantages: Increased physical activity.  Disadvantage: No benefit from the method. |
| 31 | Bell 2022 | Exercise therapy is a type of physical activity that is recommended and advanced to meet particular therapeutic objectives. | 28; 3384 | Advantages: Findings suggest that combining resistance training with education aimed at enhancing pain management and self-efficacy may, in the medium term, have a greater impact on physical activity than education alone.  Disadvantages: Adiposity or blood measurements were not found in the trials that were included. |
| 32 | Hirohama 2023 | Aerobic structured land-based exercises | 4; 256 | Advantages: Educational interventions for physical activity are an effective way to reduce knee pain, even in situations where physical contact is difficult, such as during the COVID-19 pandemic.  Disadvantages: The effect was not so significant. |
| 33 | Hamada 2022 | (group) educational intervention for improving pain | 4; 423 | Advantages: Exercise does not involve personalized instruction, Significant pain reduction after the group educational intervention, effectiveness of group lessons in preserving independence, minimizing knee discomfort, and preventing impairment in older persons.  Disadvantages: It is challenging to definitively conclude that the group educational intervention by itself had a substantial impact on reducing knee pain. |
| 34 | Runge 2022 | Aerobic exercise, strengthening exercises, stretching exercises, proprioception exercises, balance training, neuromuscular training, and functional training | 19; 1394 | The study discovered that manual therapy as an adjuvant to exercise therapy offers modest short-term advantages on pain and the WOMAC global scale in patients with hip or knee OA discomfort. Manual treatment, on the other hand, did not provide long-term benefits when used in conjunction with exercise therapy for individuals with hip or knee OA. Before adopting manual therapy as an additional therapy, clinicians should first focus on the basic interventions of exercise and education. When discussing their management plan with hip or knee OA patients, clinicians should clearly emphasize the absence of long-term advantages of adopting manual therapy in addition to exercise. |
| 35 | Yang 2022 | Strengthening exercises, combined with stretching, aerobic exercises | 9; 861 | Advantages: Exercise therapies based on telehealth are useful for knee osteoarthritis: Comparable to in-person care; Significantly superior to standard care; Long-term pain treatment (>3 months) can be improved with web or smartphone apps; Rural delivery improves access for patients who live in rural areas or have limited mobility; Convenient for individuals who have difficulty traveling.  Disadvantages: Consider target population preferences as well as technological limits. Consider phone interventions for rural places and the elderly. Larger sample size research is required (several small and pilot experiments are included). Recognize that heterogeneity in rcts (different exercise modalities, intervention length, and frequency) influences total results. |
| 36 | Wen 2022 | Tai Chi, yoga, and qigong | 17; 1481 | Advantages: In terms of the benefits of mind-body exercise, the study finds that Tai Chi has been clinically demonstrated to increase self-efficacy and physical function in senior patients, reduce the risk of falls, and have a good influence on blood pressure regulation. Tai Chi has also been included into cognitive-behavioral therapy to decrease stress in people with human immunodeficiency virus infection, as well as the health management of fibromyalgia patients. Tai Chi training appears to be beneficial to chronic KOA pain and CLBP in the chronic pain population. The study produced comparable results and compensated for the limitations of these meta-analyses' inadequate sample size.  Disadvantages: Concerning the study's shortcomings, such as the difficulties in blinding patients and coaches during mind-body workout treatments, which may add to potential risks of performance bias, the study recognizes some. Furthermore, some of the studies included in the study failed to include critical data such as random sequence generation, intention to treat analysis, and allocation concealment, which could have resulted in an overestimation of the pooled effect size. Overall, while mind-body exercise shows promise as a non-pharmacological intervention for chronic pain, further study is needed to overcome these limitations and fully understand the benefits and drawbacks of this treatment. |
| 37 | Migliorini 2022‌ | The study investigated the type of sport and activity level of athletes before they underwent knee and/or hip arthroplasty for osteoarthritis (OA), but did not categorize the types and modes of exercise into cardio, flexibility, or resistance | 5; 3638 | Does not provide a direct comparison of the advantages and disadvantages |
| 38 | Thorlund 2022 | Exercise therapy | 13+101; 1398 | According to the study, the benefits and drawbacks of the methodology utilized in the network meta-analysis studies were not adequately stated. The authors did remark, however, that the definition and reporting of adverse events differed, making it impossible to pool this data in a meaningful way. Furthermore, some changes from the published protocol occurred, such as the inclusion of trials with mixed populations and the use of an external anchor in the network meta-analysis that included trials comparing the three examined therapies with placebo/control interventions. The GRADE method was also employed by the authors to assess the overall quality of evidence. |
| 39 | Zeng 2021 | Traditional exercise, Tai Chi,Multimodal exercise,Aquatic exercise,Neuromuscular exercise,High-intensity interval training (HIIT),Mind-body exercise | 38; NG | Advantages: Pain and inflammation are reduced. Increased joint mobility and function. Enhanced muscular strength and endurance. Increased life satisfaction and psychological well-being  Disadvantage: Injury or worsening of symptoms if exercise is not conducted correctly or under competent supervision. Depending on their specific health issues or limits, some types of exercise may not be appropriate for all persons. Exercise may not be useful for everyone who has knee osteoarthritis, and additional treatments or interventions may be required. |
| 40 | Hall 2021‌ | Strengthening, mixed, mind-body, and stretching | 21; NG | Does not provide a direct comparison of the advantages and disadvantages of different exercise programs for psychological well-being in knee osteoarthritis. The study focused on comparing the effectiveness of different types of exercise programs for improving mental health outcomes in people with knee osteoarthritis. |
| 41 | Goff 2021 | The types and modes of exercise used in the included studies may have varied, but the study did not specifically focus on evaluating the effectiveness of different types or modes of exercise | 29; 4107 | Does not provide information on the advantages or disadvantages of this method compared to other interventions or treatments. The study focused on evaluating the effectiveness of patient education interventions combined with exercise therapy in improving pain and function in people with knee osteoarthritis, but it did not compare these interventions to other methods or report any disadvantages associated with them. |
| 42 | You 2021 | Tai Chi exercise | 11; 603 | Advantages:  According to this source, the benefits of Tai Chi exercise for senior people with knee osteoarthritis include: Tai Chi has been shown to be highly beneficial in improving the walking abilities and posture control of older people with knee osteoarthritis, according to this source, as indicated by both self-reported and experimental data. Tai Chi is a low-impact, low-risk exercise choice for older persons suffering from knee osteoarthritis, especially when practiced under the supervision of a skilled instructor. Tai Chi is suitable for home-based practice since it can be done comfortably in one's own house, making it a practical exercise option for elderly persons who may have mobility issues or limited access to outdoor facilities. Potential benefits for joint flexibility, blood circulation, and respiratory function: Tai Chi has been shown to improve joint flexibility, blood circulation, and respiratory function, potentially contributing to improved general health and immune system function.  Disadvantages: There are no downsides to Tai Chi exercise for senior people with knee osteoarthritis, according to the source. |
| 43 | Raposo 2021 | Strengthening and aerobic exercise | 19; 1126 | Advantages: According to the systematic review presented on this page, exercise therapies have various advantages for treating knee osteoarthritis. These include pain relief, enhanced physical function, and an overall higher quality of life. Exercise therapies can also help improve knee joint stability and lower the incidence of long-term knee injury.  While exercise therapies are generally safe and beneficial for treating knee osteoarthritis, they can induce mild problems such as muscle soreness, joint stiffness, and weariness. These difficulties, however, are usually transient and can be controlled by making suitable changes to the workout routine. Furthermore, some patients may find it difficult to stick to an exercise regimen for a variety of reasons, including a lack of enthusiasm, time limits, or physical limitations. |
| 44 | Grantham 2021 | Leg-press exercise, strength training, flexibility exercises and balance training | 5; 199 | The potential of BFRT to enhance muscle strength and growth with lower loads may be advantageous for persons with knee OA who are unable to tolerate high loads due to discomfort or joint deterioration. Furthermore, BFRT may be a less time-consuming and less expensive alternative to traditional resistance training. The report does, however, state that more research is needed to determine the long-term effects of BFRT on knee OA patients and to discover any potential dangers or disadvantages connected with this training regimen. |
| 45 | Chen 2020 | Types and modes of exercise are not specified, but it is said that they were individual and could be provided remotely. | 12; NG | Advantages: Significant knee pain reduction after short-term implementation of the programs. Technology-supported exercise programs can be customized to the needs of people with knee OA or persistent knee pain. Remote delivery of technology-supported exercise programs increases accessibility and convenience for people who find it challenging to attend in-person sessions. Real-time feedback on exercise performance provided via technology-supported exercise programs increase motivation and program adherence.  Disadvantages: It is hard for some people to use particular forms of technology because of cognitive, physical, or behavioral limitations. Some technologically assisted exercise programs such as virtual reality or game-based exercise treatment, can be costly and require technical help from medical specialists. There were several limitations to the review studies, such as the possibility of clinical variation among people with knee OA or persistent knee pain, which might lead to statistical heterogeneity in the meta-analysis. |
| 46 | Luan 2020 | Stationary cycling exercise, stretching, strengthening, Tai Chi, and Baduanjin. | 8; NG | Advantages: Stationary cycling exercise helps people with knee ostencing function and quality of life. Oarthritis by lowering pain and enha. Stationary cycling is a low-impact type of exercise that is simple to adapt to a person's demands and physical capabilities. Since stationary cycling can be done indoors, it is a practical choice for people who do not have access to outdoor exercise facilities or who want to exercise in a regulated setting. |
| 47 | Kawabata 2020 | Muscle strengthening exercises and knee stability training.  Quadriceps strengthening, hamstring curls, heel lifts, and agility-perturbation training | 7; NG | Does not provide information on the advantages or disadvantages of this method compared to other interventions or treatments. |
| 48 | Li 2020 | Tai Chi and Baduanjin. | 14; 815 | The advantages of TCE for KOA patients include its potential efficacy in lowering symptoms including pain, stiffness, and physical function, as well as its generally safe and well-tolerated nature with no serious side effects recorded in the majority of the included trials. However, there are publishing and linguistic biases that might lead to an overestimation of TCE intervention effectiveness. The interpretation of these results may also have been affected by the included rcts' methodological shortcomings. To further establish the efficacy and underlying mechanisms of TCE for treating KOA, further high-quality rcts with a large sample size and long-term interventions are needed. |
| 49 | Rocha 2020 | Resistance and endurance training, aerobic and balance exercises, | 7; 934 | Exercise has advantages for treating knee osteoarthritis, including reduced pain, increased muscular strength, and enhanced proprioception. Another inexpensive, non-invasive therapy option that may be readily incorporated into a patient's everyday routine is exercise. The length and level of exercise necessary to produce the best benefits are not yet clearly understood, and the absence of a gold standard tool for measuring muscular strength may make it difficult to analyze this variable quantitatively. Additionally, due to a paucity of research on these effects, it is unknown what the long-term advantages of exercise treatment and potential prevention of osteoarthritis will be. |
| 50 | Hu 2020 | Tai Chi training forms, such as Chen style, Yang style, Wu Hao style, Wu style, and Sun style | 16; 986 | Tai Chi exercises can considerably lessen patients' emotional symptoms including fear of falling, despair, and anxiety in addition to their physical symptoms. Given the advantages for patients, the lack of additional expense, the unique location, and independence from the effects of the weather, Tai Chi practice should be actively encouraged for patients with knee osteoarthritis as part of the rehabilitation management program. |
| 51 | Hall 2020 | Used in reviewed studies: isotonic and isometric strengthening exercises,combination of aerobic and resistance exercises, cornerstone treatment | 18; NG | Advantages: Improved pain and physical function outcomes. Possibility of long-term advantages, since exercise programs can result in long-lasting reductions in pain and function. Exercise therapies are typically safe for people with knee osteoarthritis, therefore there is a low chance of negative side effects. |
| 52 | Van Doormaal 2020 | A combination of muscle strengthening, aerobic, and neuromuscular exercises. | NG; NG | Advantages: Non-pharmacological therapies have benefits, such as being typically well-tolerated and safe, with few side effects reported, and being successful in lowering pain and enhancing physical function.  Disadvantages: Exercise therapy or manual therapy may cause brief increases in pain or discomfort in certain individuals during or after those treatments. Medication side effects and dangers can be considerable when taking pharmacological therapies such nonsteroidal anti-inflammatory medicines (nsaids) and opioids, especially in older persons or patients with comorbidities. |
| 53 | Zampogna2020 | Land-based exercise: strength training, aerobic exercise, and combined exercise programs.  Aquatic exercise:  Hydrotherapy, pool exercise, and swimming.  Sports: Tai Chi, Yoga, and Baduanjin. Different modes of exercise: group exercise, home-based exercise, and supervised exercise. | 22; NG | Advantages: Physical activity has been proven to be particularly good for older adults with knee and hip OA in terms of pain relief, improved function, performance, and quality of life, with statistically significant improvements compared to the control group. Patients with hip and knee osteoarthritis who were 65 years of age or older were included in the study, which examined various therapies from 22 studies that matched the inclusion criteria. A meta-analysis of 19 publications was also conducted as part of the study, indicating that a sizable number of patients were taken into account.  Disadvantages: Taking into account the various rates of adherence and adverse events, it was not able to identify with certainty whether one form of physical exercise had more long-term advantages than the others. |
| 54 | Schulz 2019 | Exercise programs: aerobic, strength, and neuromuscular | 14+10; NG | Advantages: The study included quantitative analysis to assess how therapies including aerobic exercise affected indicators of systemic inflammation and cardiovascular health. The FITT principle, a commonly used framework for creating exercise regimens, was used in the study to provide the exercise recommendations. The study discovered that therapies involving aerobic exercise can enhance cardiovascular health and lower systemic inflammation in people with knee osteoarthritis.  Disadvantages: The majority of the included studies were found to have a significant bias-related risk, which could compromise the reliability of the findings. According to GRADE, the study's evidence quality was low, hence it is best to proceed with care when interpreting the findings. The research made no mention of any information on problems or adverse effects related to the exercise regimens. |
| 55 | Vitaloni 2019 | Education and supervised exercise | 62; NG | Advantages: The study adhered to the Preferred Reporting Items for Systematic Reviews and Meta-Analyses (PRISMA) standards, which are regarded as the industry's benchmark for systematic reviews. To find relevant studies, the article employed a comprehensive search technique that encompassed surgical and non-surgical data and did not impose any restrictions on the comparative control group. The quality of the studies that were utilized in the review was evaluated by the article using a quality rating technique.  Disadvantages: A meta-analysis of the papers evaluated was absent from the report, which would have allowed for a more quantitative overview of the results. The review's repeatability may be hampered by the paper's lack of a thorough explanation of the search approach employed. The paper's lack of a thorough explanation of the quality assessment technique might reduce the review's openness. |
| 56 | Verhagen 2019 | Types and modes of exercise are not specified | 42; 6863 | Does not mention any specific advantages/disadvantages of the method. |
| 57 | Goh 2019 | Resistance: strengthening exercises  Flexibility: tai chi | 77; 6472 | Advantages include a considerable reduction in pain, improvement in function, performance, and overall quality of life. These positive effects are particularly notable after an 8-week period.  Disadvantages: The advantages associated with exercise gradually decrease over a period of time, resulting in outcomes that are comparable to standard treatment after 9 to 18 months. The potential decrease in exercise adherence with time may contribute to the observed drop in efficacy. |
| 58 | Kraus 2019 | Resistance: strengthening exercises.  Cardio: aerobic exercises,  Flexibility: tai chi | 261; 25924 | Advantages: Physical activity decreased pain, improved physical function and health-related quality of life among people with hip or knee OA. Also, the benefits of physical activity persisted for up to 6 months after stopping a defined program.  Disadvantages: There's a potential U-shaped or J-shaped dose–response relationship of physical activity with OA progression for those with preexisting OA. |
| 59 | Chen 2019 | Cardio: aquatic exercises | 6; 432 | There are several advantages associated with this approach. Firstly, it allows for a comprehensive search across different databases, ensuring a thorough examination of relevant literature. Additionally, the inclusion of randomized controlled trials (rcts) enhances the rigour and reliability of the findings. Lastly, the utilisation of known outcome measures such as the Western Ontario and mcmaster Universities Osteoarthritis Index (WOMAC) and the Knee injury and Osteoarthritis Outcome Score (KOOS) ensures consistency and comparability in assessing outcomes.  Drawbacks: The existing data is limited, thus requiring additional research. |
| 60 | Imoto 2019 | Resistance: strengthening exercises,  Proprioception exercises;  Cardio: aerobic exercises,  Flexibility: tai chi and yoga | 55; NG | Advantages: The synthesis considered a mix of different exercise types, emphasizing not just strengthening but also other types of exercises. This synthesis can be a pragmatic instrument for health professionals to get information about effective exercises for knee OA.  Disadvantages: The outcome of this study is solely focused on pain, not considering other outcomes like physical function or quality of life. Concerns over the risk of bias assessment in the included studies. High heterogeneity among the studies. Variations in the control group across the studies, which might affect the effect size. Execution of home-based exercises in some studies is questionable due to adherence issues |
| 61 | Goh 2019 | Cardio: aerobic  Flexibility: tai chi, yoga.  Resistance: strengthening exercises | 103; 9134 | Advantages: This NMA was designed to examine the relative efficacy between exercises specifically for knee and hip OA. Included a larger number of trials (103) compared to previous nmas. Used a different exercise classification based on the ACSM criteria. Examined four outcomes (pain, self-reported function, observed performance, and qol) while the previous review examined only two.  Disadvantages: Reliance on author descriptions for classification of exercises and control groups. Exercise programs and 'usual care' are not standardized and vary between studies. Estimates for some exercises were open to considerable uncertainty due to a small number of studies. Focus of the included studies was short-term and mainly single-joint OA. |
| 62 | Hislop 2020 | Resistance: hip strengthening,  Functional neuromuscular, quadriceps exercises | 8; 341 | Advantages: Provides evidence for clinicians to consider the type of hip exercises prescribed for people with KOA to enhance outcomes over quadriceps alone. Offers insights into the potential benefits of hip resistance exercises.  Disadvantages: Quality of evidence was rated very low. Inconsistent results across studies. Follow-up time points were short-term (3 months or less). Limited to radiological diagnosis of KOA of mild-to-moderate severity. Inadequate reporting on exercise details, adherence, and instructor qualifications. |
| 63 | Dong 2018 | Resistance: land-based, aerobic | 8; 579 | Advantages: AQE has many advantages compared to LBE and is recommended for post-total knee arthroplasty patient rehabilitation. AQE showed a higher level of adherence and satisfaction compared to LBE.  Disadvantages: Lack of effectiveness might be attributed to the heterogeneity of the included studies. Variations in exercise prescription across the studies. Different water temperatures and depths in AQE could directly impact exercise outcomes. Only a small number of studies with limited sample size were included. Absence of detailed descriptions of exercise programs. Lack of blinding due to the nature of the interventions. |
| 64 | Kus 2019 | Resistance: Quadriceps Femoris Muscle (QFM). | 10; 759 | Advantages: The majority of the studies incorporated in the analysis exhibit a substantial degree of evidence, methodological rigor, and minimal risk of bias.  There are some drawbacks associated with this research. Firstly, the heterogeneity of study designs poses a challenge in terms of comparing and synthesizing the findings. Additionally, the low number of available studies may restrict the comprehensiveness and generalizability of the results. Lastly, the inclusion criteria of only considering papers published in English may introduce a language bias, potentially excluding relevant research conducted in other languages. |
| 65 | Raghava 2020 | Muscle strengthening exercises, specifically hip muscle strengthening.  Included exercises such as: side-lying and standing hip abduction and adduction, side-lying clam, unilateral or bilateral leg press, standing hip flexion and extension, seated hip external, and internal rotation, among others. | 5; 331 | Advantages: One of the notable advantages of this study is its ability to synthesize and consolidate the existing literature pertaining to the topic, thereby offering a thorough and exhaustive set of findings. The use of home-based rehabilitation programs can effectively facilitate the sustained provision of treatment over an extended period. A high level of adherence to hip muscle-strengthening exercise was noted.  There are several disadvantages and limitations associated with this phenomenon. The intervention exhibited heterogeneity within the research groups in terms of the specific hip muscles targeted, the intensity of exercise programs, and the duration of these programs. The determination of the optimal dosage of the workout program was not achieved. The physical activity levels of the individuals were not taken into account in any of the research. None of the studies included in the analysis examined the cost-effectiveness of the intervention. |
| 66 | Van Ginckel 2019 | Aerobic exercise (specifically walking)  Strengthening exercises: weight-bearing (closed chain) and non-weight-bearing (open chain) resistance exercises for major lower and/or upper limb muscle groups.  Neuromuscular exercise: weight-bearing functional exercises emphasizing performance quality.  High-impact multidirectional weight-bearing aerobic or step-aerobic jumping exercises. | 7; NG | One of the advantages of this study is its comprehensive systematic review and meta-analysis. The GRADE technique was employed to assess the quality of evidence. The inclusion of MRI outcomes allows for a more comprehensive examination of cartilage and structural alterations.  There are several drawbacks associated with this phenomenon. The study's findings were characterized by a lack of directness and precision, as well as a limitation to relatively inaccurate effect estimates in individuals with an average obesity status. The generalizability of the findings is somewhat constrained as they are only relevant to individuals with knee osteoarthritis who are, on average, fat. Based on investigations conducted over a period of 17 or 18 months, it is arguable that the duration of these research may be insufficient to comprehensively examine alterations in radiographic illness. A limited number of papers met the criteria for inclusion in the meta-analysis. |
| 67 | Bricca 2018 | The specific types of exercise are not mentioned in the provided abstract. However, the context suggests varying intensities and durations of exercise. | 3; NG | One of the advantages of this study is that it adhered to the requirements set forth by the Preferred Reporting Items for Systematic Reviews and Meta-Analyses. The Grading of Recommendations evaluation, Development, and Evaluation (GRADE) approach was employed to conduct a quality evaluation.  One of the drawbacks identified in this study is the low overall quality of data, which indicates the necessity for further high-quality randomized controlled trials (rcts). |
| 68 | Bricca 2019 | Knee joint loading exercise | 9; NG | One of the advantages of this study is that it adhered to the Preferred Reporting Items for Systematic Reviews and Meta-analyses (PRISMA) criteria.  There are several drawbacks associated with this phenomenon. The level of evidence presented exhibited a low quality. The presence of heterogeneity in the interventions, patient characteristics, and outcome variables. Certain studies have reported a lack of adherence to exercise treatments. The studies did not have the opportunity to compare various workout routines or examine specific cartilage compartments. A meta-analysis was not conducted. |
| 69 | Schäfer 2018 | Home therapy, exercise, healthy diet, pain management, and self-management | 6; 742 | Not mentioned |
| 70 | Hurley 2018 | Land-based or aquatic-based exercise program aiming to improve OA symptoms | 21; 2372 | In the absence of proper guidance from healthcare professionals, individuals may lack clarity about what's safe and beneficial, leading them to avoid physical activity due to fear of potential harm. Engaging in exercise programs can lead to minor improvements in physical function, alleviate depression and pain to some extent. It may also slightly enhance self-efficacy and social function. However, the evidence suggests that there is likely only a minimal or negligible impact on anxiety. |
| 71 | Young 2018 | Eccentric or heavy, slow resistance exercises | 59; 583 | The text describes the dosing of exercises, without providing straight comparison. However, for knee osteoarthritis, 24 exercise sessions and 8- and 12- week durations of it were parameters most often associated with large effects.  An exercise frequency of once per week was associated with no effect. |
| 72 | Kanavaki 2017 | The main focus of the paper was on barriers and facilitators of physical activity in general. Authors did not divide exercises. | 51; 5449 | Barriers: Fatigue and stiffness, obesity, doubtful effectiveness  Facilitators: Pain relief, joint stability, accurate knowledge |
| 73 | Umehara 2018 | The main focus of the paper was on the periods of starting activity in general. Authors mentioned exercises as  Knee joint flexion and extension. Measurements used to assess body function (or impairment) varied and included pain, physical function, stiffness, muscle strength, and ROM | 27; 2432 | Advantages: Exercise starting after discharge in addition to standard postoperative interventions was led by improved flexion ROM and required at least 8 weeks.  Disadvantages: Starting exercises in hospital does not affect the result |
| 74 | Minshull 2017 | Strengthening, weight lifting, muscle contraction, using resistance training machines, free weights, ankle weights, sandbags, elastic resistance bands, and isometric holds | 34; 1574 | Advantages: Increasing the resistance of the exercise to maintain 80% 1 RM, which was measured. Biweekly, 30 a 5% biweekly increment of resistance of the initial 50% 1 RM  Disadvantages: Limitations in treatment prescription and patient adherence. |
| 75 | Fernandopulle 2017 | Tai chi/ Baduajin, walking, and conditioning exercise,  Martial arts | 27; NG | Advantages: A significant improvement in pain scales in the short-term; A significant improvement in the WOMAC physical function subscale for the intervention group; Significant improvement in VAS scores.  Disadvantages: Exacerbation, injuries sustained because of trips/fall while participating in the intervention program. |
| 76 | Brosseau 2017 | Resistance: strengthening exercises | 26; NG | Advantages: The research offers suggestions with a hierarchical alphabetical grading system, so facilitating comprehension of the significance and therapeutic relevance of the findings.  Disadvantages: One potential drawback is to the limited availability of data about the extended-term sustainability of frequent strengthening exercise regimens. |
| 77 | Brosseau 2017 | Cardio: aerobic exercise | 5; NG | Not mentioned |
| 78 | Brosseau 2017 | Hatha Yoga,  Tai Chi Qigong,  Sun style Tai Chi. | 4; NG | Advantages: One advantage of the proposed approach is the utilization of a hierarchical alphabetical grading system that is founded on both statistical significance and clinical value. The systematic search was employed, and the selection criteria were modified accordingly. A Delphi survey was conducted, involving a panel of experts who achieved consensus. |
| 79 | Zhang 2017 | Tai Chi, Baduanjin | 8; 375 | Advantages: Quality of life and mental health.  Disadvantages: Rest of the meta-analysis did not change the results. |
| 80 | Nicolson 2017 | Aerobic exercise, strengthening, flexibility, balance or body-region- specific exercises | 9; 1045 | Advantages: It is possible for the effectiveness of booster sessions, and indeed other interventions designed to increase adherence.  Disadvantages: A slight behavior change intervention. |
| 81 | Maly 2016 | Cycling, swimming, Resistance | 18; 1860 | Advantages: Patients who received exercise treatment showed lower radiographic degeneration than a group of patients who experienced operative treatment. Compared to surgical methods, neuromuscular training showed improvements in muscle strength, dynamic stability, postural awareness, and muscle coordination. Research also suggested that one of the advantages of exercise treatment of ACL injuries is ist showing low risk of osteoarthritis. It is also worth mentioning that exercise treatment is way more affordable than operative treatment.  Disadvantages: Individuals who experienced exercise treatment had poorer stability and subjective function scores than a group of people who underwent surgery. Moreover, there is evidence that exercise may increase the risk of knee instability and subsequent meniscus damage, which can lead to the high-level risk of developing knee osteoarthritis. Moreover, compared to the surgical methods, conservative approach is harder to conduct some evaluations of its effectiveness of exercise. |
| 82 | Timmins 2017 | Cardio, running | 15; 3576 | Advantages: According to a meta-analysis of case-control studies, running reduces the level of risk of knee OA surgery. Moreover, studies on sclerosis rates and joint surfaces show that running has a positive impact on musculoskeletal health. Also, running, a common form of physical activity, improves overall fitness and cardiovascular health.  Disadvantages: The author notes that despite the many benefits of running, the relationship between running and knee OA is still unclear because there are mixed data and assumptions about the diagnostic results of these methods. Moreover, some studies show baseline radiographic differences in patients who practise running, but the clinical significance of these differences is unclear. It is noted that running can lead to various types of unstable performance, so research on this topic is suggested for further research. |
| 83 | Deasy 2016 | Resistance | 5; 237 | Advantages: The review reports that learning more about hip strength deficits in people with knee osteoarthritis has the potential to improve training interventions and guide targeted rehabilitation approaches. Moreover, assessing hip strength in the clinical setting could play a critical role in guiding treatment strategies for knee osteoarthritis.  Disadvantages: The review did not distinguish between different stages of the disease or consider gender differences, limiting the applicability of the conclusions. Some studies included in the review demonstrated deficiencies in assessor blinding and the presence of potential confounding variables that could introduce bias. |
| 84 | Henriksen 2016 | According to a recent systematic review, current evidence does not support high-intensity exercise regimens for knee osteoarthritis (OA). Therefore, current guidelines simply state that engaging in “any form of exercise” is effective for managing knee OA pain. | 54; 9806 | Advantages: According to the study, its results allow personalized therapeutic choices. If exercise is not an option, patients may consider oral analgesics for pain management, aiding in tailored care. Moreover, this study reveals that exercise and oral analgesics are equally effective for knee osteoarthritis pain, informing health care decisions.  Disadvantages: Research lacks information on the best types, intensity, frequency, and duration of exercise for knee osteoarthritis pain, making it difficult to prescribe individualized exercise programs. Further research is needed to determine the optimal exercise for different situations and groups. Moreover, this study did not examine the potential harm of exercise or oral painkillers for knee osteoarthritis. Exercise is generally safe, but exercise studies require more attention to potential side effects and their documentation. There is limited evidence supporting the safety of exercise compared to oral analgesics, highlighting the need for further investigation in this area. |
| 85 | Coudeyre 2016 | Resistance, Isokinetic Muscle Strengthening, Isometric and Dynamic Exercise | 9; 696 | Advantages: IMS improves muscle strength, physical function, and quality of life in individuals with knee OA. IMS allows personalized treatment while tailoring to the patients’ needs and abilities. IMS uses specialized equipment, such as isokinetic dynamometers which help to measure muscle strength properly and track the progress periodically.  Disadvantages: IMS may not be appropriate for all individuals with knee osteoarthritis, particularly for those who have significant joint damage or medical conditions that restrict their exercise capacity. IMS could be expensive compared to alternative types of exercises, since it requires specialized equipment and trained professionals. Incorrect execution or insufficient warm up of muscles before IMS may lead to muscle injuries. |
| 86 | Gay 2016 | The study doesn’t include specific information about types of exercises. | 13 + 8; NG | Advantages: Providing information about physical activity and exercise increases the desire to be treated and increases the exercise and weight loss regimen, resulting in long-term benefits. Exercise and weight loss can improve functionality and relieve pain in people with osteoarthritis of the hip and knee. Self-government programs including training have proven to be effective in reducing visits to health institutions and related costs over a period of 12 months.  Disadvantages: It can be difficult to achieve the necessary changes in the patient's behavior to perform the exercise and lose weight. Programs adapted to a specific patient phenotype are needed, which may require additional resources and time from doctors. |
| 87 | Bartels 2016 | Flexibility, Resistance, strength, Cardio, aerobics | 13; 1190 | Advantages: According to reviews, water exercises bring many benefits to people suffering from osteoarthritis of the knee and hip joints. It can improve the functioning of the body, relieve pain and improve the overall quality of life. The advantage of aquatic exercises is that it is a type of low-trauma physical activity that produces less pressure on the joints. In addition, it can be adapted to improve neuromuscular control of the lower extremities, potentially reducing the negative effects of osteoarthritis.  Disadvantages: However, the review also pointed out that a limited number of trials have been conducted to evaluate long-term effects, indicating that the observed lack of efficacy may be due to the rarity of the trial rather than its inherent lack of efficacy. Based on the currently available evidence, the optimal balance between aquatic and terrestrial exercises is still uncertain. |
| 88 | Forestier 2016 | Not mentioned | 19; 1735 | Advantages: Spa therapy for knee osteoarthritis offers benefits such as pain relief, improved function, and improved quality of life.  Advantages: However, there are disadvantages such as the cost of treatment, the need to move to a spa facility, and the lack of data on the long-term effectiveness of spa treatment. |
| 89 | Rooij 2016 | Not mentioned | 15; NG | Advantages: Finds factors predicting deterioration in pain and physical functioning.  Disadvantages: Interpreting the course of pain and physical function is challenging due to the varying results of the study. Limited evidence and scarce high-quality studies make it difficult to identify predictive factors. |
| 90 | Tanaka 2015 | Not mentioned | 12; 1239 | Advantages: Exercise therapy, evaluated by the SF-36 questionnaire, improved the quality of life of patients suffering from general health-related knee osteoarthritis by improving several scores. Exercise therapy has surpassed control interventions by having a positive impact on the physical and mental components of quality of life. Exercise therapy effectively improves the overall health-related quality of life, including mental well-being, and not just the disease-specific aspects.  Disadvantages: The specific disadvantages or risks of exercise therapy for knee osteoarthritis are not mentioned in the sources. Individual factors and precautions, such as appropriate technique, appropriate intensity and monitoring by a qualified health professional, are essential to minimize possible side effects. Although exercise therapy offers significant benefits for knee osteoarthritis, consultation with a doctor or physiotherapist is essential for personalized advice and safety during treatment. |
| 91 | Regnaux 2015 | Not mentioned | 6; 656 | The review did not provide specific information about advantages and disadvantages of high- versus low-intensity physical activity or exercise programs for osteoarthritis. However, the authors emphasized that the effectiveness of exercise and physical activity depends on poorly defined components, and the health benefits can be more pronounced with high intensity than with low intensity efforts. |
| 92 | Ferreira 2015 | Resistance, aerobic, Flexibility | 3; 233 | Advantages: Exercise therapy for knee arthritis offers benefits such as improving pain control, physical functions, as well as muscle strength and torque.  Disadvantages: Notably, the review did not mention any disadvantages associated with exercise therapy. |
| 93 | Runhaar 2015 | Muscle strength and flexibility | 94; NG | Advantages: Reduced pain, decreased joint inflammation, improved joint function, increased muscle strength, maintains a healthy weight, enhanced mental well-being.  Disadvantages: risk of injury, initial discomfort and initially increased pain, requires additional time and effort, requires individual approach. |
| 94 | Anwer 2016 | A combination of cardio, flexibility, resistance training, including: kinematic chain exercises stretching, range of motion exercises, proprioception exercises, walking, tai chi exercises. | 19; NG | Advantages: convenience and flexibility, cost-effective, independence, long-term adherence and sustained improvements  Disadvantages: lack of supervision and risk of exacerbating symptoms; limited feedback, lack of self-motivation and accountability, requires individual approach. |
| 95 | Tanaka 2016 | Flexibility training, aerobic training, upper and lower body training, open-kinetic chain resistance training program, tai chi training | NG; NG | Advantages: systematic method of assessment, several factors are included, a standardized framework, informed decisions.  Disadvantages: subjective assessment, dependence on availability of evidence, potential for bias and systematic errors, time-consuming process. |
| 96 | Lu 2015 | Aquatic exercises, aerobic exercises, range of motion exercises, strength exercises, and balance exercises. | NG; 398 | Advantages: comprehensive analysis, high reliability and low potential for bias, quantitative synthesis, high statistical power, generalizability to larger population.  Disadvantages: dependence on availability of evidence, publication bias, heterogeneity, challenging quality assessment, challenging causality determination |
| 97 | Quintrec 2014 | Cardio, resistance and aquatic exercises. | 13; NG | Advantages: reduced pain, adequate tolerance, designated approach.  Disadvantages: lack of persistence, potential side effects, low reliability of the method. |
| 98 | Uthman 2013 | Strengthening exercises, flexibility exercises and cardio. | 60; 8218 | Advantages: extensive comparison, great statistical power, ranked interventions, insights on heterogeneity.  Disadvantages: potential for bias, inconsistency between evidences, lack of the data, challenging interpretation. |
| 99 | Ye 2014 | Exercises with elements of Tai Chi, that are focused on muscle strength, flexibility and cardio. | 6; 309 | Advantages: relieves pain, improves the balance, systematic review, robust study design, variety of databases.  Disadvantages: limited number of studies, lack of up-to-date information. |
| 100 | Tanaka 2014 | Resistance, strengthening exercises and cardio | 17; NG | Advantages: reduces pain.  Disadvantages: individual approach is required |
| 101 | Henriksen 2014 | Cardio, walking | 5; 452 | Advantages: provides comprehensive summary on the available data, assess the reliability of the data, involves different resources (cohort studies and controlled trials)  Disadvantages: lack of data, limited number of studies. |
| 102 | Waller 2014 | Aquatic exercises, cardio, resistance | 11; 1092 | Advantages: one of the common treatment methods, enhanced pain control and physical performance, low dropout rate and high persistence.  Disadvantages: side effects, such as increased pain, difficult to assess the long-term effects, lack of data and small sample size. |
| 103 | Juhl 2014 | Cardio, resistance, performance exercises | 48; 4028 | Advantages: provides a comprehensive summary, suggests an optimal exercise regimen, analyses the specific exercise characteristics.  Disadvantages: dependence on availability and quality of data, differences in the studies, no side effects mentioned. |
| 104 | Kroman 2014 | Cardio, performance exercises (walking, hop) | 20; NG | Advantages: provides a comprehensive summary, uses standardized tools, such as COSMIN to evaluate the quality of characteristics, includes different measurement properties, conducts a targeted assessment of measures.  Disadvantages: lack of data, no information on hip populations, limited generalizability, lack of updated information. |
| 105 | Tanaka 2013 | Resistance, aerobic exercise. | 8; NG | Advantages: The impact of confounding variables on the effectiveness of exercise is taken into account in the study. It also considers the effects of exercise frequency and duration on the efficacy of exercise.  Disadvantages: The study has several limitations, including the quality of trials included. The meta-analysis included trials with low quality, indicating a high risk of bias. There is also a potential issue of publication bias. |
| 106 | Tanaka 2013 | Resistance, aerobic exercise; walking,: Tai chi; Badu Anjin; range of motion exercise. | 33; 3,192 | Advantages: Objective result data that has been combined from several investigations. The degree of evidence for the different kinds of results was revealed.  Disadvantages: The investigations exhibited variations in subject characteristics. The presence of statistical heterogeneity was noted across different outcomes in the studies. |
| 107 | Wang 2012 | Aerobic exercise, aquatic exercise, Tai Chi, Strengthening exercise, proprioception exercise. | 193; NG | Advantages: The study conducted a thorough examination, incorporating a substantial quantity of randomized controlled trials (rcts), with a particular emphasis on outcomes that prioritize the well-being and preferences of patients.  Disadvantages: The lack of consistency in physical therapy treatments and outcome measurements has impeded the capacity to effectively synthesize research. The presence of inconsistencies in the definitions and measures of outcomes. Most trials conducted have focused on evaluating individual therapies, resulting in limited availability of information about the effectiveness of integrated physical therapy interventions. |
| 108 | Dobson 2012 | Study mentions performance-based measures related to walking tests (over short  Distances (<100 m) and over long distances), sit to stand tests, and stair negotiation tests, which could be categorised as cardio exercise. | 24; NG | Advantages: The study offers valuable insights for clinicians and researchers on the optimal performance-based metrics for evaluating individuals with hip and/or knee osteoarthritis.  Disadvantages: The internal validity of a study may be compromised by the presence of publication bias resulting from the exclusion of unpublished research. The choice to omit specific measures may have imposed constraints on the extent of the evaluation. Most of the evidence from several studies could not be integrated due to differences in performance-based metrics. |
| 109 | Smith 2012 | Proprioceptive exercises, Weight-bearing in nature, based on functional activities such as stepping, standing, walking, and balancing. Some used a computer program or an electrogenic, Lower limb exercises with a focus on strengthening regimes for quadriceps and hamstring muscle groups. | 7; 560 | Disadvantages: The methodological quality of the evidence base was found to be moderate, with only three studies demonstrating the use of a power calculation to determine their sample size. There exists a potential for type II statistical mistakes. |
| 110 | Silva 2012 | Aerobic exercise, Tai Chi, Strength training, hydrotherapy, vibrating platform exercise, balance exercises, strength training with controlled or uncontrolled weights, and educational programs. | 9; NG | Advantages: The high methodological rigor of the studies incorporated in this systematic review suggests that the therapeutic exercises they implemented enhanced the balance of women with knee OA.  Disadvantages: In the absence of a comprehensive procedural description, clinical findings cannot be replicated in new studies. Immediate conclusions could be drawn concerning the optimal timing and frequency of intervention, the necessary instruments, and any potential adverse effects. |
| 111 | Batterham 2011 | Aquatic exercise, Exercise training for strength, endurance, or aerobic capacity (both gym and home-based). | 10; NG | Advantages: Two independent reviewers methodically extracted data that were relevant to the review problem.  Disadvantages: Most studies had design errors, hence limiting the level of confidence in the reported results. There exists a lack of information pertaining to patient satisfaction or adherence to exercise regimens. |
| 112 | Jansen 2011 | Aerobic activity exercises, Strength training. | 12; NG | Advantages: This study presents empirical findings about the efficacy of various exercise regimens in managing pain and improving functional outcomes among individuals diagnosed with knee osteoarthritis.  Disadvantages: The evaluation offers a limited assessment of the various treatment modalities, providing only an indirect comparison and without any direct comparisons across the three intervention kinds. |
| 113 | Escalante 2011 | Aerobic programs (mainly walking-based), Mixed programs (aerobic exercises such as walking, static bicycle, or other aerobic activities, Tai chi, Resistance | 20; 2142 | Advantages: This study offers valuable information about the efficacy of different exercise programs in improving functional aerobic capacity among individuals diagnosed with hip and knee osteoarthritis.  Disadvantages: The workout programs exhibit a significant degree of heterogeneity in their structure. Many research encounters constraints or biases, which pose challenges in establishing definitive results. The extent of the individuals' osteoarthritis (OA) is documented in a limited number of investigations, namely eight. The interventions exhibit a significant degree of heterogeneity in terms of their substance. The regulation of intensity is only described in seven of the papers that were analyzed. |
| 114 | Escalante 2010 | Aerobic programs (e.g., walking, or other aerobic activities chosen by the patient), Tai Chi programs (e.g., “Sun” style, “Wu” style, “Yang” style, simplified adaptations “Baduanjin”, and “Qigong”), Resistance | 33; NG | Advantages: Not mentioned.  Disadvantages: Very few randomized clinical trials were carried out, despite recommendations for the use of exercise regimens as pain management in individuals with osteoarthritis of the hip and knee. The exercise programs ' structure is highly variable in terms of content, duration, frequency, and session length. |
| 115 | Delarue 2007 | Cardio,strength, flexibility. | 47; NG | Advantages: Combining literature review with the analysis of daily practice by French physicians.  Disadvantages: The difficulty in characterizing directed and nondirected physical therapy, the variety of programs and assessment instruments, and the absence of long-term analysis. |
| 116 | Tiffreau 2007 | Endurance training, walking, treadmill, cycling.  Passive mobilization, muscle and tendon lengthening. Static or dynamic, resistive, isokinetic, with immersion, global or analytical lower-limb muscle strengthening; strengthening resistance (weight bearing, isokinetic strengthening). | 29; NG | Advantages: The study employed the SOFMER technique, which integrates a systematic examination of the literature, collection of real-world clinical practices, and an external evaluation conducted by a diverse panel of experts.  Disadvantages: The study did not yield definitive information about the comparative advantages of either the individual or group modality. The long-term maintenance of exercise therapy's effectiveness is contingent upon the sustained continuation of the program. |

**Abbreviations**

NG – Not Given

PA – Physical Activity

MRI – Magnetic Resonance Imaging

KAM – Knee Adduction Moment

TKA – Total Knee Arthroplasty

KO – Knee Osteoarthritis

KOA – Knee Osteoarthritis

OA – Osteoarthritis

APM – Arthroscopic Partial Meniscectomy

BFRT – Blood Flow Restriction Training

RCT – Randomized Controlled Trial

TCE – Traditional Chinese Exercises

WOMAC – Western Ontario and McMaster Universities Osteoarthritis Index

KOOS – Knee injury and Osteoarthritis Outcome Score

BDNF – Brain-Derived Neurotrophic Factor

WQX – Wu Qin Xi

BCTs – Behavior Change Techniques

CLBP – Chronic Low Back Pain

HIIT – High-Intensity Interval Training

NSAIDs – Non-Steroidal Anti-Inflammatory Drugs

FITT – Frequency, Intensity, Time, Type

PRISMA – Preferred Reporting Items for Systematic Reviews and Meta-Analyses

NMA – Network Meta-Analysis

ACSM – American College of Sports Medicine

QOL – Quality of Life

AQE – Aquatic Exercise

LBE – Land-Based Exercise

QFM – Quadriceps Femoris Muscle

ROM – Range of Motion

VAS – Visual Analogue Scale

RM / 1RM – Repetition Maximum / One Repetition Maximum

IMS – Isokinetic Muscle Strengthening

COSMIN – COnsensus-based Standards for the selection of health Measurement INstruments

SOFMER – Société Française de Médecine Physique et de Réadaptation (French Society of Physical Medicine and Rehabilitation)

**Table 2.** AMSTAR-2 evaluation. “+” Yes; “PY” Partial Yes; “-“ No. Abbreviations: MA, meta-analysis; N/A, not available.

| **#** | **Author, year** | **1** | **2** | **3** | **4** | **5** | **6** | **7** | **8** | **9** | **10** | **11** | **12** | **13** | **14** | **15** | **16** |
| --- | --- | --- | --- | --- | --- | --- | --- | --- | --- | --- | --- | --- | --- | --- | --- | --- | --- |
| 1 | Cottmeyer 2023 | + | PY | + | + | + | PY | - | + | + | - | + | PY | PY | + | + | + |
| 2 | Lopes 2023 | PY | - | + | + | + | - | N/A | + | + | - | + | PY | PY | - | - | + |
| 3 | Massey 2022 | + | + | + | + | + | - | N/A | + | + | - | - | N/A | + | + | - | + |
| 4 | Sheikhhoseini 2023 | PY | + | + | + | PY | PY | + | + | + | - | + | + | + | + | + | + |
| 5 | Si 2023 | + | PY | + | + | + | + | + | PY | + | - | + | PY | PY | PY | + | + |
| 6 | Zhang 2023 | + | N/A | + | + | + | + | + | + | + | - | + | N/A | N/A | + | + | + |
| 7 | Puts 2023 | + | + | + | + | + | - | N/A | + | + | + | N/A | N/A | + | PY | PY | + |
| 8 | Chang 2023 | N/A | + | N/A | + | N/A | N/A | N/A | N/A | + | N/A | N/A | N/A | N/A | N/A | N/A | N/A |
| 9 | Mo 2023 | N/A | + | N/A | + | + | + | N/A | N/A | + | N/A | - | - | N/A | N/A | + | + |
| 10 | Chaudhry 2023 | - | + | - | + | + | - | - | N/A | + | - | - | - | N/A | N/A | - | + |
| 11 | Clausen 2023 | N/A | + | - | + | PY | N/A | + | N/A | + | - | - | - | + | N/A | + | + |
| 12 | Yokoyama 2023 | - | - | - | PY | + | + | + | - | - | + | + | - | - | - | - | + |
| 13 | Singla 2023 | + | + | - | PY | + | + | + | PY | PY | + | + | - | - | - | + | + |
| 14 | Xu 2023 | + | PY | - | PY | + | + | + | PY | PY | - | no MA | no MA | - | - | + | - |
| 15 | Malik 2023 | + | PY | - | PY | + | + | + | PY | + | - | + | - | - | + | + | - |
| 16 | Patterson 2023 | - | PY | - | PY | + | + | + | - | + | - | - | - | + | - | + | - |
| 17 | Xu 2023 | + | PY | - | PY | + | + | + | PY | - | + | + | - | - | + | - | + |
| 18 | Coburn 2022 | + | PY | - | PY | + | + | + | PY | + | - | + | + | + | + | + | + |
| 19 | Sasaki 2022 | + | PY | - | PY | + | + | + | PY | + | - | + | + | + | + | + | - |
| 20 | Guo 2022 | - | PY | - | PY | + | + | + | - | PY | - | + | + | + | + | + | + |
| 21 | Calaido 2022 | + | PY | - | PY | + | + | + | PY | + | + | no MA | no MA | - | - | no MA | + |
| 22 | Guo 2022 | + | PY | - | PY | + | + | + | PY | PY | + | + | + | + | + | + | + |
| 23 | Wu 2022 | + | + | - | PY | + | + | + | PY | PY | + | + | + | + | + | + | + |
| 24 | Ariie 2022 | + | + | - | + | + | + | + | PY | PY | + | + | + | + | + | + | - |
| 25 | Silva 2022 | + | + | - | PY | + | + | + | + | - | - | + | + | + | + | + | + |
| 26 | Granicher 2022 | + | + | - | + | + | + | + | + | - | - | + | + | + | + | + | + |
| 27 | Jurado-Castro 2022 | + | + | - | PY | + | + | + | PY | PY | + | + | + | + | + | - | + |
| 28 | Rotini 2022 | + | + | - | PY | + | + | + | PY | - | + | + | + | + | + | + | + |
| 29 | Saueressig 2022 | + | + | - | PY | + | + | + | + | - | - | + | - | - | - | + | + |
| 30 | Fernandez-Matias 2022 | + | PY | - | PY | + | + | - | + | PY | + | - | + | + | + | - | + |
| 31 | Bell 2022 | + | + | - | - | + | + | + | PY | - | + | - | - | - | - | - | + |
| 32 | Hirohama 2023 | + | PY | - | - | + | + | PY | PY | PY | + | + | + | + | - | - | - |
| 33 | Hamada 2022 | + | PY | - | - | + | + | + | PY | - | + | - | + | - | - | - | + |
| 34 | Runge 2022 | + | + | + | - | + | - | PY | PY | RCTs PY  NRSI - | - | - | + | + | + | - | - |
| 35 | Yang 2022 | + | + | + | PY | + | + | + | + | RCTs +  NRSI - | - | RCTs +  NRSI - | + | + | + | + | - |
| 36 | Wen 2022 | + | PY | + | + | + | + | + | + | RCTs PY  NRSI - | + | RCTs +  NRSI - | - | - | + | + | - |
| 37 | Migliorini 2022‌ | + | - | - | PY | + | - | + | PY | - | - | - | - | - | - | - | - |
| 38 | Thorlund 2022 | + | PY | + | PY | + | + | - | + | RCTs +  NRSI - | - | RCTs +  NRSI - | + | + | + | - | + |
| 39 | Zeng 2021 | + | - | + | - | - | - | - | - | - | - | - | - | - | - | - | + |
| 40 | Hall 2021‌ | + | PY | - | PY | - | - | - | - | - | - | - | + | + | + | - | - |
| 41 | Goff 2021 | + | + | + | + | + | + | + | + | + | - | RCTs +  NRSI - | + | + | + | + | + |
| 42 | You 2021 | - | - | + | - | - | - | - | PY | RCTs PY  NRSI - | - | RCTs +  NRSI - | + | + | + | - | - |
| 43 | Raposo 2021 | + | PY | - | PY | + | + | + | + | RCTs +  NRSI - | - | - | - | + | + | - | - |
| 44 | Grantham 2021 | + | PY | + | PY | + | + | PY | PY | RCTs +  NRSI - | - | RCTs +  NRSI - | + | + | + | - | - |
| 45 | Chen 2020 | + | - | - | PY | + | + | PY | + | + | - | + | + | + | + | + | + |
| 46 | Luan 2020 | - | + | - | PY | + | + | + | + | PY | + | + | + | - | + | - | + |
| 47 | Kawabata 2020 | + | PY | - | PY | - | - | - | PY | + | - | + | - | - | - | - | + |
| 48 | Li 2020 | + | PY | - | PY | - | - | - | + | PY | - | + | + | + | + | + | + |
| 49 | Rocha 2020 | - | - | - | - | + | + | - | + | + | - | + | + | + | + | - | - |
| 50 | Hu 2020 | + | PY | - | - | + | + | - | PY | + | - | + | + | + | + | + | - |
| 51 | Hall 2020 | + | + | - | PY | + | + | + | + | + | - | + | + | + | + | - | + |
| 52 | van Doormaal 2020 | + | + | - | - | - | - | - | + | + | - | - | - | - | - | - | + |
| 53 | Zampogna2020 | - | + | + | - | + | + | - | + | - | - | + | - | - | + | - | + |
| 54 | Schulz 2019 | - | - | - | PY | + | + | + | PY | + | - | + | + | + | + | - | - |
| 55 | Vitaloni 2019 | - | - | + | PY | + | - | + | + | - | - | N/A | N/A | + | + | N/A | - |
| 56 | Verhagen 2019 | + | + | - | PY | + | + | + | + | PY | - | - | + | + | + | + | + |
| 57 | Goh 2019 | + | PY | + | PY | + | + | + | + | + | - | + | + | + | + | + | - |
| 58 | Kraus 2019 | + | PY | + | + | + | + | + | + | + | + | + | + | + | + | - | + |
| 59 | Chen 2019 | + | PY | + | PY | + | + | + | PY | + | - | - | + | + | + | - | - |
| 60 | Imoto 2019 | + | - | + | PY | + | + | PY | + | + | + | + | + | + | + | - | + |
| 61 | Goh 2019 | + | PY | + | + | + | + | PY | + | + | + | + | + | + | + | - | - |
| 62 | Hislop 2020 | + | PY | + | PY | + | + | PY | PY | + | - | + | + | + | + | - | - |
| 63 | Dong 2018 | + | - | + | PY | + | + | + | PY | PY | - | + | + | + | + | - | - |
| 64 | Kus 2019 | + | - | + | + | + | + | + | PY | PY | - | + | - | + | - | - | - |
| 65 | Raghava 2020 | + | - | + | PY | + | + | + | PY | PY | – | - | - | + | + | - | - |
| 66 | Van Ginckel 2019 | + | PY | + | PY | + | + | PY | PY | PY | - | + | + | + | - | - | - |
| 67 | Bricca 2018 | + | - | + | - | - | - | - | - | - | - | + | + | + | + | - | - |
| 68 | Bricca 2019 | + | PY | + | PY | + | + | PY | PY | PY | - | - | - | + | - | - | - |
| 69 | Schäfer 2018 | + | + | - | PY | + | + | - | PY | +- | - | +- | + | + | + | + | - |
| 70 | Hurley 2018 | + | + | + | + | + | + | + | + | ++ | - | ++ | + | + | + | + | - |
| 71 | Young 2018 | + | - | + | + | + | + | - | + | ++ | - | no MA | + | + | + | - |  |
| 72 | Kanavaki 2017 | - | + | + | + | + | + | - | + | + PY | - | no MA | no MA | + | - | no MA | - |
| 73 | Umehara 2018 | + | - | + | + | + | + | + | + | ++ | - | + | + | + | + | - | - |
| 74 | Minshull 2017 | + | + | + | PY | + | + | + | + | -+ | - | - - | - | + | + | - | - |
| 75 | Fernandopulle 2017 | + | + | + | + | + | + | + | + | + + | - | + + | + | + | + | - | - |
| 76 | Brosseau 2017 | + | PY | + | + | + | + | + | + | + - | - | + - | + | + | + | - | - |
| 77 | Brosseau 2017 | + | + | + | + | + | + | + | + | + - | - | + - | + | + | + | - | - |
| 78 | Brosseau 2017 | + | + | + | + | + | + | + | - | + - | - | + - | + | + | - | - | - |
| 79 | Zhang 2017 | + | - | + | + | + | + | + | + | + + | + | + - | + | + | + | + | - |
| 80 | Nicolson 2017 | + | - | + | + | + | + | + | + | + + | - | + - | + | + | + | + | - |
| 81 | Maly 2016 | + | PY | - | PY | + | + | PY | PY | - | - | no MA | no MA | - | - | no MA | - |
| 82 | Timmins 2017 | + | + | - | PY | + | + | + | PY | PY | - | + | - | - | + | + | + |
| 83 | Deasy 2016 | + | + | - | PY | + | + |  | PY | PY | - | + | - | - | + | + | - |
| 84 | Henriksen 2016 | + | + | - | PY | + | + | + | PY | + | + | + | + | - | + | - | + |
| 85 | Coudeyre 2016 | + | + |  | PY | + | + | PY | PY | - | - | + | - | - | + | - | - |
| 86 | Gay 2016 | + | + | - | PY | + | + | PY | PY | + | - | + | + | + | - | - | + |
| 87 | Bartels 2016 | + | + | - | + | + | + | + | PY | + | + | + | + | + | + | + | + |
| 88 | Forestier 2016 | + | + | - | PY | + | + | + | PY | + | + | + | + | + | + | + | - |
| 89 | Rooij 2016 | + | + | - | PY | + | + | + | PY | - | - | + | + | + | + | - | - |
| 90 | Tanaka 2015 | + | + | - | PY | + | + | + | PY | - | - | + | - | - | + | + | - |
| 91 | Regnaux 2015 | + | + | - | PY | + | + | + | PY | + | + | + | + | + | + | + | - |
| 92 | Ferreira 2015 | + | PY | - | PY | + | + | + | PY | + | - | + | + | + | + | + | - |
| 93 | Runhaar 2015 | + | PY | - | + | + | + | - | PY | RCTs: -  Includes only RTCs | + | no MA | no MA | - | - | no MA | + |
| 94 | Anwer 2016 | + | + | + | + | + | + | - | PY | RCTs: + | + | RCTs: + | + | + | + | + | + |
| 95 | Tanaka 2016 | + | + | + | + | + | + | - | PY | RCTs: PY | + | RCTs: + | + | + | + | + | + |
| 96 | Lu 2015 | + | + | + | + | + | + | + | + | RCTs: + | + | RCTs: + | + | + | + | - | + |
| 97 | Quintrec 2014 | + | PY | - | PY | - | - | - | + | RCTs: PY | - | no MA | no MA | - | - | no MA | + |
| 98 | Uthman 2013 | + | + | + | + | + | + | - | PY | RCTs: + | + | RCTs: + | + | + | + | + | + |
| 99 | Ye 2014 | + | PY | + | + | + | + | - | + | PY | - | no MA | no MA | - | - | no MA | - |
| 100 | Tanaka 2014 | + | + | - | PY | + | + | - | PY | RCTs: PY | - | + | + | + | + | + | - |
| 101 | Henriksen 2014 | + | + | + | + | + | + | - | + | RCTs: - | + | RCTs: + | + | + | + | + | + |
| 102 | Waller 2014 | + | + | + | + | + | + | - | PY | RCTs: + | - | RCTs: + | + | + | + | + | - |
| 103 | Juhl 2014 | + | + | + | + | + | + | - | PY | RCTs: + | + | RCTs: + | + | + | + | + | - |
| 104 | Kroman 2014 | + | - | - | + | + | + | - | + | - | + | no MA | no MA | - | - | no MA | + |
| 105 | Tanaka 2013 | + | + | + | PY | + | + | + | + | RCTs:  - | - | RCTs: +  NRSI: - | + | + | + | + | - |
| 106 | Tanaka 2013 | + | PY | + | PY | + | + | + | PY | RCTs:+ | - | RCTs: +  NRSI: - | + | + | + | - | - |
| 107 | Wang 2012 | + | + | + | PY | + | + | + | + | RCTs: + | + | RCTs: + | + | + | + | - | + |
| 108 | Dobson 2012 | + | + | + | PY | + | + | + | PY | RCTs: +  NRSI: PY | - | no MA | no MA | + | + | no MA | + |
| 109 | Smith 2012 | + | PY | + | + | + | + | + | + | RCTs: PY | - | RCTs: +  NRSI: - | + | + | + | - | - |
| 110 | Silva 2012 | + | PY | + | PY | + | + | + | + | RCTs: PY | - | no MA | no MA | + | + | no MA | - |
| 111 | Batterham 2011 | + | PY | - | PY | + | + | + | - | RCTs: + | - | RCTs: +  NRSI: - | + | + | + | - | - |
| 112 | Jansen 2011 | + | - | + | - | + | + | + | + | RCTs: PY  NRSI: PY | - | RCTs: +  NRSI: - | + | + | + | - | + |
| 113 | Escalante 2011 | + | + | + | PY | + | + | + | - | RCTs: +  NRSI: - | + | RCTs: +  NRSI: - | + | + | + | - | - |
| 114 | Escalante 2010 | + | - | + | - | + | + | + | PY | RCTs: PY  NRSI: - | + | RCTs: -  NRSI: - | + | + | + | - | - |
| 115 | Delarue 2007 | + | PY | + | - | + | + | - | + | RCTs: - | - | RCTs: -  NRSI: - | - | + | - | - | - |
| 116 | Tiffreau 2007 | + | PY | + | - | + | - | - | PY | RCTs: -  NRSI: - | - | no MA | no MA | + | + | no MA | - |

1. Did the research questions and inclusion criteria for the review include the components of PICO?
2. Did the report of the review contain an explicit statement that the review methods were established prior to the conduct of the review and did the report justify any significant deviations from the protocol?
3. Did the review authors explain their selection of the study designs for inclusion in the review?
4. Did the review authors use a comprehensive literature search strategy?
5. Did the review authors perform study selection in duplicate?
6. Did the review authors perform data extraction in duplicate?
7. Did the review authors provide a list of excluded studies and justify the exclusions?
8. Did the review authors describe the included studies in adequate detail?
9. Did the review authors use a satisfactory technique for assessing the risk of bias (RoB) in individual studies that were included in the review?
10. Did the review authors report on the sources of funding for the studies included in the review?
11. If MA was performed did the review authors use appropriate methods for statistical combination of results?
12. If MA was performed, did the review authors assess the potential impact of RoB in individual studies on the results of the MA or other evidence synthesis?
13. Did the review authors account for RoB in individual studies when interpreting/ discussing the results of the review?
14. Did the review authors provide a satisfactory explanation for, and discussion of, any heterogeneity observed in the results of the review?
15. If they performed quantitative synthesis did the review authors carry out an adequate investigation of publication bias (small study bias) and discuss its likely impact on the results of the review?
16. Did the review authors report any potential sources of conflict of interest, including any funding they received for conducting the review?
