# Supplementary material for: Impact of therapeutic exercises on pain-related outcomes in patients with knee osteoarthritis: an umbrella review of 116 systematic reviews
Source: Front Pain Res (Lausanne). 2026 Mar 10;7:1717540. doi: 10.3389/fpain.2026.1717540 (PMC13008949; doi:10.3389/fpain.2026.1717540)
Supplement: Supplementary file 2 [file Supplementaryfile2.docx]

**Table 3.** Reported Outcomes, Evidence Direction, and Quality Ratings of Included Articles

|  | **Author, country, year** | **Reported outcomes** | **Direction** | **Quality of the article** |
| --- | --- | --- | --- | --- |
| 1 | Cottmeyer 2023 | Pain, Knee biomechanics | Pain – Positive  Knee biomechanics – Insignificant | Low |
| 2 | Lopes 2023 | Pain, Knee biomechanics | Pain – Positive, Knee biomechanics - Positive | Critically low |
| 3 | Massey 2022 | Adherence rate | Adherence rate - Insignificant | Critically low |
| 4 | Sheikhhoseini 2023 | Knee biomechanics | Knee biomechanics - Positive | High |
| 5 | Si 2023 | Pain, Physical function, Quality of life | Pain – Positive, Physical function – Positive, Quality of life - Positive | High |
| 6 | Zhang 2023 | Pain, Physical function | Pain – Insignificant, Physical function - Positive | High |
| 7 | Puts 2023 | Pain | Pain - Positive | High |
| 8 | Chang 2023 | Pain, Physical function | Pain – Positive, Physical function - Positive | High |
| 9 | Mo 2023 | Pain, Physical function, Quality of life | Pain – Positive, Physical function – Positive, Quality of life - Positive | Low |
| 10 | Chaudhry 2023 | Physical function, Quality of life | Physical function – Positive, Quality of life – Insignificant | Critically low |
| 11 | Clausen 2023 | Severity of the disease | Severity - Inconclusive | Low |
| 12 | Yokoyama 2023 | Pain, Knee biomechanics | Pain – Positive, Knee biomechanics - Inconclusive | Critically low |
| 13 | Singla 2023 | Flexion range of motion | Flexion range of motion - Positive | Low |
| 14 | Xu 2023 | Pain, Knee biomechanics | Pain – Positive, Knee biomechanics – Positive | Low |
| 15 | Malik 2023 | Pain | Pain - Positive | Low |
| 16 | Patterson 2023 | Knee biomechanics | Knee biomechanics - Positive | Low |
| 17 | Xu 2023 | Pain, Physical function, Knee biomechanics | Pain – Positive, Physical function – Positive, Knee biomechanics - Positive | Critically low |
| 18 | Coburn 2022 | Knee biomechanics | Knee biomechanics - Inconclusive | Moderate |
| 19 | Sasaki 2022 | Pain, Knee biomechanics | Pain – Positive, Knee biomechanics - Inconclusive | Moderate |
| 20 | Guo 2022 | Pain | Pain - Positive | Moderate |
| 21 | Calaido 2022 | Functional capacity | Functional capacity - Positive | Low |
| 22 | Guo 2022 | Pain, Physical function | Pain – Positive, Physical function - Positive | High |
| 23 | Wu 2022 | Pain | Pain - Positive | High |
| 24 | Ariie 2022 | Pain, Physical function, Quality of life | Pain – Insignificant, Physical function – Inconclusive, Quality of life – Inconclusive | Moderate |
| 25 | Silva 2022 | Knee biomechanics | Knee biomechanics - Inconclusive | Low |
| 26 | Granicher 2022 | Knee biomechanics | Knee biomechanics - Positive | Low |
| 27 | Jurado-Castro 2022 | Pain, Physical function, Quality of life | Pain – Positive, Physical function – Positive, Quality of life - Positive | Low |
| 28 | Rotini 2022 | Pain | Pain - Positive | Low |
| 29 | Saueressig 2022 | Knee biomechanics | Knee biomechanics - Positive | Critically low |
| 30 | Fernandez-Matias 2022 | Pain, Quality of life, Knee biomechanics | Pain – Insignificant, Quality of life – Insignificant, Knee biomechanics - Inconclusive | Critically low |
| 31 | Bell 2022 | Knee biomechanics | Knee biomechanics - Positive | Critically low |
| 32 | Hirohama 2023 | Pain | Pain - Positive | Critically low |
| 33 | Hamada 2022 | Pain | Pain - Positive | Critically low |
| 34 | Runge 2022 | Pain | Pain - Positive | Critically low |
| 35 | Yang 2022 | Pain, Physical function, Quality of life | Pain – Positive, Physical function – Positive, Quality of life – Insignificant | Moderate |
| 36 | Wen 2022 | Pain, Physical function, Quality of life | Pain – Positive, Physical function – Positive, Quality of life – Insignificant | Low |
| 37 | Migliorini 2022‌ | Risk of arthroplasty | Risk of arthroplasty – Positive (this is the worsening) | Critically low |
| 38 | Thorlund 2022 | Pain | Pain - Positive | Critically low |
| 39 | Zeng 2021 | Pain, Knee biomechanics | Pain – Positive, Knee biomechanics - Positive | Critically low |
| 40 | Hall 2021‌ | Mental well-being | Mental well-being - Positive | Critically low |
| 41 | Goff 2021 | Pain | Pain - Positive | High |
| 42 | You 2021 | Knee biomechanics | Knee biomechanics - Inconclusive | Critically low |
| 43 | Raposo 2021 | Pain, Physical function, Quality of life, Knee biomechanics | Pain – Positive, Physical function – Positive, Quality of life – Positive, Knee biomechanics - Positive | Critically low |
| 44 | Grantham 2021 | Pain, Physical function, Knee biomechanics | Pain – Insignificant, Physical function – Insignificant, Knee biomechanics - Inconclusive | Low |
| 45 | Chen 2020 | Pain, Physical function, Quality of life | Pain – Positive, Physical function – Positive, Quality of life - Positive | Low |
| 46 | Luan 2020 | Pain, Physical function, Quality of life | Pain – Positive, Physical function – Positive, Quality of life - Positive | Critically low |
| 47 | Kawabata 2020 | Knee biomechanics | Knee biomechanics - Positive | Critically low |
| 48 | Li 2020 | Pain, Physical function | Pain – Positive, Physical function - Positive | Low |
| 49 | Rocha 2020 | Pain, Physical function, Knee biomechanics | Pain – Positive, Physical function – Positive, Knee biomechanics - Positive | Critically low |
| 50 | Hu 2020 | Pain, Physical function, Quality of life | Pain – Positive, Physical function – Positive, Quality of life - Positive | Critically low |
| 51 | Hall 2020 | Pain, Physical function | Pain – Positive, Physical function - Positive | Low |
| 52 | Van Doormaal 2020 | Pain, Physical function | Pain – Positive, Physical function - Positive | Critically low |
| 53 | Zampogna2020 | Pain, Physical function, Quality of life | Pain – Positive, Physical function – Positive, Quality of life - Positive | Critically low |
| 54 | Schulz 2019 | Systemic inflation | Systemic inflation - Inconclusive | Critically low |
| 55 | Vitaloni 2019 | Quality of life | Quality of life - Positive | Critically low |
| 56 | Verhagen 2019 | Pain | Pain - Positive | Low |
| 57 | Goh 2019 | Pain, Physical function, Quality of life | Pain – Positive, Physical function – Positive, Quality of life - Positive | Moderate |
| 58 | Kraus 2019 | Pain, Physical function, Quality of life | Pain – Positive, Physical function – Positive, Quality of life - Positive | Low |
| 59 | Chen 2019 | Pain, Physical function, Quality of life, Knee biomechanics | Pain – Insignificant, Physical function – Insignificant, Quality of life – Insignificant, Knee biomechanics - Inconclusive | Critically low |
| 60 | Imoto 2019 | Pain | Pain - Positive | Critically low |
| 61 | Goh 2019 | Pain, Physical function, Quality of life | Pain – Positive, Physical function – Positive, Quality of life - Positive | Low |
| 62 | Hislop 2020 | Pain, Quality of life | Pain – Positive, Quality of life – Insignificant | Low |
| 63 | Dong 2018 | Pain, Physical function, Quality of life | Pain – Insignificant, Physical function – Positive, Quality of life – Insignificant | Critically low |
| 64 | Kus 2019 | Pain, Physical function | Pain – Positive, Physical function - Positive | Critically low |
| 65 | Raghava 2020 | Pain, Physical function, Knee biomechanics | Pain – Positive, Physical function – Positive, Knee biomechanics - Positive | Critically low |
| 66 | Van Ginckel 2019 | Knee biomechanics | Knee biomechanics - Positive | Low |
| 67 | Bricca 2018 | Knee biomechanics | Knee biomechanics - Positive | Critically low |
| 68 | Bricca 2019 | Knee biomechanics | Knee biomechanics - Positive | Critically low |
| 69 | Schäfer 2018 | Physical function, Quality of life | Physical function – Positive, Quality of life - Positive | Low |
| 70 | Hurley 2018 | Pain, Physical function | Pain – Positive, Physical function - Positive | Moderate |
| 71 | Young 2018 | Pain, Physical function | Pain – Positive, Physical function - Positive | Critically low |
| 72 | Kanavaki 2017 | Self-efficacy | Self-efficacy - Inconclusive | Low |
| 73 | Umehara 2018 | Pain, Physical function, Knee biomechanics | Pain – Positive, Physical function – Positive, Knee biomechanics - Inconclusive | Critically low |
| 74 | Minshull 2017 | Knee biomechanics | Knee biomechanics - Inconclusive | Critically low |
| 75 | Fernandopulle 2017 | Pain, Physical function | Pain – Positive, Physical function - Positive | Low |
| 76 | Brosseau 2017 | Pain, Physical function, Quality of life | Pain – Positive, Physical function – Positive, Quality of life - Positive | Low |
| 77 | Brosseau 2017 | Pain, Physical function, Quality of life | Pain – Positive, Physical function – Positive, Quality of life - Positive | Low |
| 78 | Brosseau 2017 | Pain, Physical function, Quality of life | Pain – Positive, Physical function – Positive, Quality of life - Positive | Low |
| 79 | Zhang 2017 | Pain, Physical function, Quality of life | Pain – Positive, Physical function – Positive, Quality of life - Positive | Low |
| 80 | Nicolson 2017 | Adherence rate | Adherence rate – Positive (Moderate) | Low |
| 81 | Maly 2016 | Risk of osteoarthritis | Risk of osteoarthritis - Inconclusive | Low |
| 82 | Timmins 2017 | Running | Running - Positive | Low |
| 83 | Deasy 2016 | Hip strength deficit | - | Low |
| 84 | Henriksen 2016 | Pain | Pain - Positive | Critically low |
| 85 | Coudeyre 2016 | Pain, Physical function, Quality of life, Knee biomechanics | Pain – Positive, Physical function – Positive, Quality of life – Positive, Knee biomechanics - Inconclusive | Critically low |
| 86 | Gay 2016 | Pain, Physical function | Pain – Positive, Physical function - Positive | Low |
| 87 | Bartels 2016 | Pain, Quality of life | Pain – Positive, Quality of life - Positive | High |
| 88 | Forestier 2016 | Pain, Physical function, Quality of life | Pain – Positive, Physical function – Positive, Quality of life - Positive | Moderate |
| 89 | Rooij 2016 | Pain, Physical function | Pain – Insignificant, Physical function - Inconclusive | Critically low |
| 90 | Tanaka 2015 | Pain, Quality of life | Pain – Positive, Quality of life - Positive | Critically low |
| 91 | Regnaux 2015 | Pain, Physical function | Pain – Insignificant, Physical function - Positive | Moderate |
| 92 | Ferreira 2015 | Pain, Physical function, Knee biomechanics | Pain – Positive, Physical function – Positive, Knee biomechanics - Inconclusive | Moderate |
| 93 | Runhaar 2015 | Pain, Knee biomechanics | Pain – Positive, Knee biomechanics - Inconclusive | Low |
| 94 | Anwer 2016 | Knee biomechanics | Function - Positive | Low |
| 95 | Tanaka 2016 | Walking | Walking - Positive | Low |
| 96 | Lu 2015 | Pain, Physical function, Quality of life | Pain – Insignificant, Physical function – Positive, Quality of life – Insignificant | Low |
| 97 | Quintrec 2014 | Pain | Pain - Positive | Critically low |
| 98 | Uthman 2013 | Pain | Pain - Positive | Low |
| 99 | Ye 2014 | Pain, Physical function | Pain – Positive, Physical function - Positive | Critically low |
| 100 | Tanaka 2014 | Pain | Pain - Positive | Low |
| 101 | Henriksen 2014 | Knee joint loading | Knee joint loading - Insignificant | Critically low |
| 102 | Waller 2014 | Pain, Physical function, Quality of life | Pain – Positive, Physical function – Positive, Quality of life - Positive | Low |
| 103 | Juhl 2014 | Pain | Pain - Positive | Low |
| 104 | Kroman 2014 | Performance | - | Critically low |
| 105 | Tanaka 2013 | Pain | Pain - Positive | Moderate |
| 106 | Tanaka 2013 | Pain, Knee biomechanics | Pain – Positive, Knee biomechanics - Positive | Low |
| 107 | Wang 2012 | Pain | Pain - Positive | Low |
| 108 | Dobson 2012 | Physical function | Physical function - Inconclusive | High |
| 109 | Smith 2012 | Pain, Physical function, Quality of life, Knee biomechanics | Pain – Insignificant, Physical function – Positive, Quality of life – Insignificant, Knee biomechanics - Inconclusive | Low |
| 110 | Silva 2012 | Pain | Pain - Positive | Moderate |
| 111 | Batterham 2011 | Function | Function - Insignificant | Low |
| 112 | Jansen 2011 | Pain, Physical function | Pain – Positive, Physical function - Positive | Critically low |
| 113 | Escalante 2011 | Aerobic capacity | Aerobic capacity - Inconclusive | Low |
| 114 | Escalante 2010 | Therapy programs | - | Critically low |
| 115 | Delarue 2007 | Pain | Pain - Insignificant | Critically low |
| 116 | Tiffreau 2007 | Pain | Pain - Positive | Critically low |
